# Resting-state functional connectivity of the cerebellum-cerebrum in older women with depressive symptoms

**DOI:** 10.1186/s12888-023-05232-7

**Published:** 2023-10-10

**Authors:** Lanling Feng, Dongmei Wu, Shaolun Ma, Li Dong, Yuchuan Yue, Tao Li, Yixun Tang, Zixiang Ye, Guoju Mao

**Affiliations:** 1 Nursing Department, The Clinical Hospital of Chengdu Brain Science Institute, MOE Key Laboratory for Neuroinformation, University of Electronic Science and Technology of China, Chengdu, China; 2 The Clinical Hospital of Chengdu Brain Science Institute, MOE Key Laboratory for Neuroinformation, University of Electronic Science and Technology of China, Chengdu, China

**Keywords:** Functional magnetic resonance imaging, Cerebellar-cerebral functional connectivity, Older women, Depressive symptoms

## Abstract

**Background:**

Although there has been much neurobiological research on major depressive disorder, research on the neurological function of depressive symptoms (DS) or subclinical depression is still scarce, especially in older women with DS.

**Objectives:**

Resting-state functional magnetic resonance imaging (rs-fMRI) was used to compare functional connectivity (FC) between the cerebellum and cerebral in older women with DS and normal controls (NC), to explore unique changes in cerebellar FC in older women with DS.

**Methods:**

In all, 16 older women with DS and 17 NC were recruited. All subjects completed rs-fMRI. The 26 sub-regions of the cerebellum divided by the AAL3 map were used as regions of interest (ROI) to analyze the difference in FC strength of cerebellar seeds from other cerebral regions between the two groups. Finally, partial correlation analysis between abnormal FC strength and Geriatric Depression Scale (GDS) score and Reminiscence Functions Scale (RFS) score in the DS group.

**Results:**

Compared with NC group, the DS group showed significantly reduced FC between Crus I, II and the left frontoparietal region, and reduced FC between Crus I and the left temporal gyrus. Reduced FC between right insula (INS), right rolandic operculum (ROL), right precentral gyrus (PreCG) and the Lobule IX, X. Moreover, the negative FC between Crus I, II, Lobule IX and visual regions was reduced in the DS group. The DS group correlation analysis showed a positive correlation between the left Crus I and the right cuneus (CUN) FC and GDS. In addition, the abnormal FC strength correlated with the scores in different dimensions of the RFS, such as the negative FC between the Crus I and the left middle temporal gyrus (MTG) was positively associated with intimacy maintenance, and so on.

**Conclusion:**

Older women with DS have anomalous FC between the cerebellum and several regions of the cerebrum, which may be related to the neuropathophysiological mechanism of DS in the DS group.

**Supplementary Information:**

The online version contains supplementary material available at 10.1186/s12888-023-05232-7.

## Introduction

Depressive symptoms are when a person shows signs of depression, such as sadness, anxiety and despair, but does not yet meet the clinical diagnostic criteria for depression, and are a precursor and transitional state to depression [1].The number of older people with DS is much higher than those who are clinically diagnosed. According to previous studies, the prevalence rate of DS in the older is as high as 37.52%. [2]. Female gender, old age, widowhood, low economic status, and physical illness are important risk factors for DS [3]. Studies have shown that long-term DS can develop into depression, which can lead to adverse outcomes such as cardiovascular disease, disability, cognitive impairment and suicide [4–6]. In addition, Diniz et al. point out that DS is an important risk factor for all-cause mortality in older people [7]. In recent years, with the development and application of fMRI, depression, as a common and serious mental health problem in older women, has been the subject of in-depth study of its cerebral network mechanism [8]. Previous neuroimaging studies have found that the cerebellum structure and function of depression patients are abnormal [9, 10], suggesting that the cerebellum may have a role in the development of depression. However, the FC of the cerebellum in older women with DS has not been systematically investigated.

Neuroimaging studies have shown that the cerebellum is the key region for emotional processing [11, 12]. Cerebellum involvement in emotional processing may involve several specific and non-specific regions [13]. Baumann found that different subregions of the cerebellum are selectively involved in different major emotions [12]. The activity of the cerebellar vermis (Lobules VI–IX) is associated with several major emotions (happiness, anger, disgust, fear, and sadness). Disgust, sadness, and happiness were associated with earthworm lobular VIIIA [14], while anger was associated with activating lobular IX. Similarly, a meta-analysis also showed the presence of emotion-related activity in Crus I, Crus II, VIII, IX, and Lobule VI of cerebellar vermis [15]. Some studies have shown that both positive and negative emotions are processed through the cerebellum [16, 17],while other studies have suggested that the processing of negative emotions is dominant [18, 19]. Neuroimaging studies have shown that negative emotions are associated with activity in left lobular VI, right lobular IV/V and bilateral Crus I [20–22], whereas positive emotions are associated with activity in right lobular VI [15, 21, 23].

Modern neuroimaging studies have shown that there is extensive FC between the cerebellum and cerebrum, and closed circuits with different functions for different cerebellar subregions [24, 25]. The abnormality of the functional connection between the brain and the cerebellum is usually associated with emotional and cognitive disorders and is considered to be one of the neurobiological changes in depression [26]. Alalade et al. showed that the connection between the cerebellar vermis and the posterior cingulate cortex may be related to the emotional management of senile depression [27]. Wenbin Guo et al. suggested that abnormalities in the fibers of the cerebellum and cerebrum in the resting state may be the basis for the pathogenesis of refractory depression [28]. Xu LY and colleagues found that the severity of depression in patients is correlated with alterations in cerebellar gray matter structure [8].Zhu Daomin et al. found a decrease in cerebellar-cerebral dynamic connectivity related to the emotional limbic network in the cerebellar subregion of depression patients compared to healthy people [8]. Ma et al. believed that the changes in the resting state of the cerebellar-cerebral could hopefully be used as a classification characteristic to distinguish depression patients from healthy people [29].

Reminiscence is a behavioral process that recalls the past experiences of individuals, this process may be intentional or unintentional, including the recollection of special or ordinary life events, these events may have been retained in memory or forgotten, but the individual recollections are accompanied by a sense of real scene reproduction [30]. Reminiscence can be experienced alone or shared with others. Different reminiscences have different functions, which is why some researchers have a classification of the functions of memories. At present, Webster’s classification of reminiscence functions has been accepted by most researchers, which divides reminiscence into 8 functions: (a) problem-solving (PS), (b) identity (ID), (c) death preparation (DP), (d) bitterness revival (BR), (e) intimacy maintenance (IM), (f) teaching (TE), (g) *boredom reduction (BD)*, (h) conversation (CO), and Webster’s developed a self-assessment questionnaire for reminiscence function, the RFS was a good psychometric properties [31]. Philippe studied older people using the RFS questionnaire and found that different reminiscence functions had different effects on physical and mental health [32]. They therefore reorganized and classified memory functions. Positive reminiscence functions (ID, PS, and DP) had positive effects on physical and mental health, negative reminiscence functions (BR, IM, and BD) had negative impacts on physical and mental health, prosocial reminiscence functions (CO and TE) had indirect impacts on physical and mental health.

Since then, more and more studies have used the RFS to examine the relationship between different reminiscence functions and mental health. Many studies had shown that older people with DS had more negative memories [33–35]. Korte and colleagues [36] reported in their study that BR and BD were positively correlated with DS. Results from a longitudinal study suggest that positive reminiscence function predicts lower levels of DS [37]. In addition, the reminiscence-based psychological intervention has been widely used with older people with depression, and reminiscence-based intervention has been shown to have a significant effect on improving DS in older people [38, 39]. The results of previous research suggest that reminiscence plays an important role in the development of DS, but there is no research that explores the cerebral-neural mechanism of memory that improves DS.

Despite many neurobiological studies of major depressive disorder, research on the neurological function of DS or subclinical depression remains scarce, especially in older women with DS. The study of DS will help to better identify potential neurobiological markers and predict the development of depression. This will provide information for treatment and prevention interventions. In this study, we used rs-fMRI to compare the FC between the cerebellum and cerebrum in older women with DS and NC. The aim was to investigate unique changes in cerebellar FC in older women with DS and to explore the relationship between the FC of different cerebral regions and the severity of DS and reminiscence functions.

## Materials and methods

### Participants

The participants were older women who had lived for a long time in communities in Chengdu city, China. All subjects were divided into DS group and NS group. The DS group inclusion criteria: (1) Aged > 60 years, who had lived in Chengdu city, China for more than 20 years; (2) *The GDS scores range from 11 to 25 (mild to moderate depressive symptoms);* (3) Communication is barrier-free; (4) Right-handers; (5) informed consent and voluntary participation in this study. The exclusion: (1) T1-weighted MRI showed cerebral infarction (lacunar infarction > 15 mm) or other vascular injuries; (2) People with serious physical illness; (3) Mini-Mental State Examination (MMSE) score ≤ 17 in the illiterate group, ≤ 20 in the primary school group, ≤ 24 in the secondary school group; (4) *Previous or current psychiatric disorder;*(5) History of psychotropic drug or alcohol abuse within 2 months;(6) During the period of study, participated in other research projects. Both groups had the same inclusion and exclusion criteria., except that the GDS score for the NC group was ≤ 10. According to the above criteria, a total of 33 subjects were enrolled (16 DS and 17 NS). All subjects had no contraindications to scanning and all participated in rs-fMRI scanning.

The GDS was used to assess DS in older women. *The GDS included 30 items and the total score was 30, with ≤ 10 being defined as no DS, 11–25 as mild or moderate DS, and ≥ 26 as severe depression* [40]. Reminiscence functions were measured using the RFS, which consists of 43 items, each scored on a scale of 1–6, the higher socres indicated stronger reminiscence functions for the dimension. [26].

Sichuan University Ethics Review Board reviewed and approved the study. the study is done in the accordance with the Declaration of Helsinki. Each participant provided written informed consent to accomplish this study after a complete description of the protocol.*This study was registered in the Chinese Clinical Trial Registry (ChiCTROCC-14,004,510)*.

### MRI acquisition and pre-processing

MRI images were acquired by Siemens Trio 3.0T magnetic resonance scanning system (Trio 3 T, Siemens Healthcare, Erlangen Germany). All subjects were scanned with three-dimensional (3D) sagittal plane high-resolution T1-weighted structural images. Functional imaging scan of the blood oxygen level-dependent (BOLD) signal at rest using the Echo Planar Imaging (EPI) sequence. The structural images were scanned using the 3DSPGR sequence, imaging parameters: repetition time/echo time (TR/TE) = 2250/2.62ms, field of view (FOV) = 256*256mm, thickness/gap = 1/0mm, volumes number = 170. Resting-state BOLD fMRI data were acquired using the EPI. The parameters were as follows: TR/TE = 2000/30ms, thickness/gap = 3/0mm, flip angle (FA) = 90°, FOV = 240*240mm, the localization images were consistent with the structural images. SPM12 was used to pre-process the rs-fMRI images (https://www.fil.ion.ucl.ac.uk/spm/), the image pre-processing included Realignment, Slice timing, Coregister, Ormalise and Smooth steps. The first 5 images were removed to ensure the subjects were in a stable state during scanning and the MRI magnetic field strength was stable. The resting-state image data is registered to high-resolution structural images in a registration step, and then normalised to the Montreal Neurological Institute (MNI) standard space. Spatial smoothing of images using a Gaussian smoothing kernel with a half width and height of 8 mm. Furthermore, further use the NIT toolkit (https://www.neuro.uestc.edu.cn/NIT.html) to regress 12 head motion parameter signals [41], white matter signals, cerebrospinal fluid signals, linear drift, and whole-cerebral mean signals, and then filtered using a pass-band filter of 0.01–0.08 Hz.

### Calculation of rs-FC

Pre-processed images were used to calculate seed-based resting-state FC using the REST1.8 toolkit. (http://www.restfmri.net/forum/). The 26 sub-regions of the cerebellum from AAL3 [42] atlas were used as ROI, 95–98 were the left and right cerebellar peduncles subregions, 99–112 were the left and right cerebellar Lobules, 113–120 were the cerebellar vermis part. Pearson correlation coefficients were calculated between the seed ROI time courses and the rest of the cerebral in a voxel-wise manner. The Fisher-z transform was applied to the generated image of the r-value of the correlation coefficient to make it obey a normal distribution.

### Statistical analysis

Statistical analysis of FC images was performed using the dpabi toolkit (http://rfmri.org/dpabi). Age, years of education, cerebral volume, and mean head movements were added to the general linear model as covariates. To investigate the difference in resting-state FC between NC and DS, the one-sample t-test was conducted for two groups to identify its significant functional network, and the differences in FC between DS and NC were analysed using the two-sample t-test. To adjust for multiple comparisons at the cluster level and control the false positive rate, the statistical map threshold was set at z > 2.32, with voxel correction at p < 0.01 and cluster correction at p < 0.05, using Gaussian random field (GRF) for z-statistics.

Following two-sample t-test results, mean value of zFC from peak t-value neighboring voxels (edge connected, SPM’s default) was entered into partial correlation analyses with GDS and RFS in the DS group, with age, years of education, cerebral volume, and mean head movement as covariates. SPSS version 22.0 was used for partial correlation analyses.

## Results

### Demographic data and clinical information of participants

*Results of demographic data and clinical information of participants are listed in* Table 1. *The age, years of education and cerebral volume didn’t differ between the two groups (P > 0.05).The DS group exhibited significantly higher GDS scores compared to the NC group (P < 0.05).There were no significant differences in the scores of various dimensions of RFS between the two groups (P > 0.05), except for the DS group’s higher score in the BR dimension compared to the NC group (P < 0.05).*

**Table 1:** Demographic and clinical characteristics of two groups

	NC group(N = 17)	DS group(N = 16)	DS vs. NC *t*-values(*p*-values)
**Age (years)**	64.06 ± 4.44	63.19 ± 4.02	-0.572(0.572)
**Education(years)**	11.47 ± 1.88	9.34 ± 3.63	-2.067(0.047)
**Cerebral volume(** ***z*** **-values)**	-0.06 ± 0.74	0.06 ± 1.22	0.330(0.744)
**Mean head movement(mm)**	0.31 ± 0.16	0.36 ± 0.15	0.957(0.346)
**GDS scores**	4.06 ± 2.13	14.88 ± 3.43	10.627(<0.001)
**RFS Scores**			
DP	14.41 ± 6.87	13.44 ± 5.95	-0.421(0.677)
ID	20.53 ± 6.58	17.31 ± 4.90	-1.537(0.135)
PS	17.76 ± 5.96	17.5 ± 5.29	-0.131(0.897)
BD	15.35 ± 7.38	17.19 ± 5.08	0.801(0.429)
IM	14.00 ± 3.97	14.69 ± 3.95	0.483(0.632)
BR	10.06 ± 3.57	15.50 ± 7.03	2.741(0.010)
CO	16.88 ± 4.47	19.31 ± 2.71	1.817(0.079)
TE	16.17 ± 5.55	15.56 ± 3.62	-0.362(0.720)

Abbreviations: PS: Problem-solving; ID: Identity; DP: Death preparation; BR: Bitterness revival; IM: Intimacy maintenance; TE: Teaching; BR: Boredom reduction;

### rs-FC patterns of cerebellar subregions

The ROI voxel FC patterns of the cerebellum were significantly different between the two groups, and different structures of the cerebellum showed specific patterns of change in the DS group. Compared with the NC group, the negative FC of cerebellar Crus (95–98 cerebral regions), cerebellar Lobule (100–101、103、105–111 cerebral regions) was decreased in the visual network, and the FC was decreased in the frontoparietal network in the DS group. The negative FC between cerebellar vermis (116–120 cerebral regions) and the visual network was decreased, and the FC was also decreased in the right subcortical area (119–120 cerebral regions). The results of group differences in FC from 26 cerebellar seed ROIs were shown in Appendix 1. The following highlights the representative cerebral FC differences from left Crus I and left Crus II seed regions in the cerebellar Crus, as well as the cerebral FC differences of the representative Lobule IX of Vermis and Lobule X of Vermis seed regions in the cerebellar vermis. Partial correlation analysis was performed between the zFC value of the difference peak area and the GDS and RFS scale, and the MNI coordinates of the peak t-value area are shown in Table 2.

**Table 2:** Position of t-value peak

Anatomical description	AAL3 label	Peak t-value of cluster	MNI coordinates of peak t-value			Cluster size(voxels)
			X	Y	Z	
**ROI: Left Crus I of cerebellar hemisphere**						
Right CUN	50	4.16	12	-90	18	1126
Left inferior parietal gyrus, excluding supramarginal and angular gyri(IPL)	65	-5.31	-60	-42	45	479
Left superior frontal gyrus(SFG),dorsolateral	3	-3.88	-15	36	33	353
Left MTG	89	-5.03	-66	-51	-12	247
Right Lobule IX of cerebellar hemisphere	110	-4.48	6	-45	-45	200
**ROI: Left Crus II of cerebellar hemisphere**						
Left lingual gyrus(LING)	51	4.17	-21	-99	-15	604
IPL	65	-4.65	-45	-54	54	399
**ROI: Lobule IX of vermis**						
Right PreCG	2	-5.06	54	3	18	332
Right inferior occipital gyrus(IOG)	58	4.47	36	-90	-6	216
**ROI: Lobule X of vermis**						
Right PreCG	2	-5.20	57	3	21	349

Abbreviations: CUN: Cuneus; MTG: middle temporal gyrus; PreCG: Precentral gyrus;

#### Results of FC between left Crus I and whole cerebral voxels

**Fig. 1:**
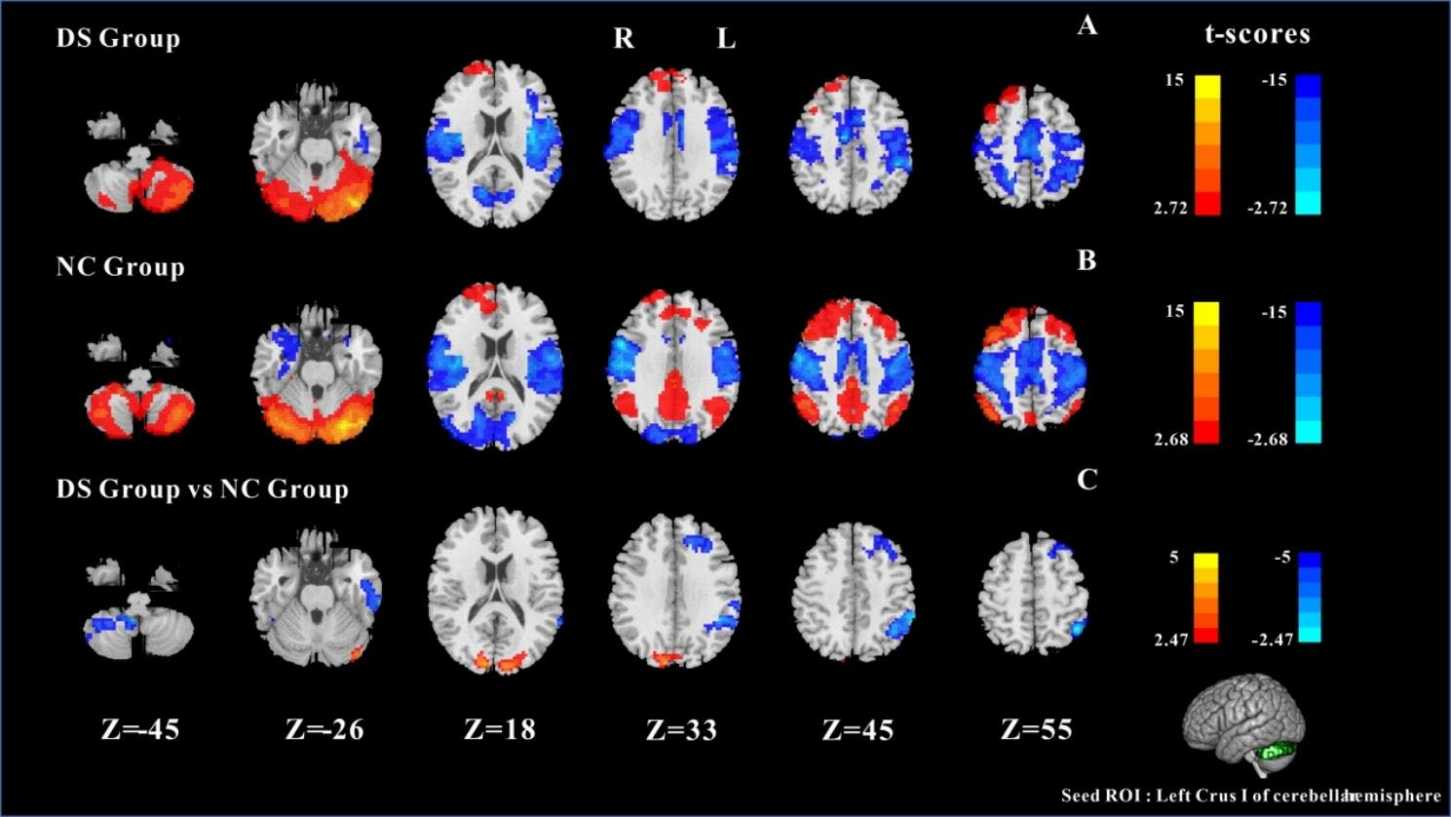
One-sample t-test results of FC based on left Crus I and whole cerebral voxels in the DS group (**A**) and in the NC group (**B**). The two-sample t-test results of FC based on left Crus I and whole cerebral voxels in the two groups (**C**); Note: the statistical map threshold was set at z > 2.32, with voxel correction at p < 0.01 and cluster correction at p < 0.05, GRF corrected for z-statistics. In one-sample t-test result, warm colors represent positive FC and cool colors represent negtive FC; In two-sample t-test result, warm colors show increased FC and cool colors show decreased FC. The color bar represents t-values. R: right, L: left

The results of the one-sample t-test of the DS and NC were shown in Fig. 1A and B, there was a significant FC between Crus I and internal cerebellum, and a significant negative FC between Crus I and sensorimotor network. In addition, within the NC group, Crus I showed significant FC with the default mode network (including the bilateral precuneus (PCUN), bilateral angular gyrus (ANG), middle posterior cingulate gyrus and bilateral SFG, and a significant negative FC with the visual area. The results of the two-sample t-test are shown in Fig. 1C, compared with NC, the DS group showed a significant increase in FC within the left Crus I cerebral region. The FC between left Crus I and right Lobule IX, left frontal parietal region (left inferior parietal gyrus (IPG), left supramarginal gyrus (SMG), left SFG, left middle frontal gyrus (MFG), and left temporal gyrus (left MTG, left inferior temporal gyrus (ITG)) decreased. Moreover, the DS group showed negative FC between the Crus I and visual region (left middle occipital gyrus (MOG), left superior occipital gyrus, right CUN, bilateral LING) was reduced. (GRF corrected, cluster *p*-value < 0.05,voxel *p-*value<0.01).

#### Results of FC between left Crus II and whole cerebral voxels

**Fig. 2:**
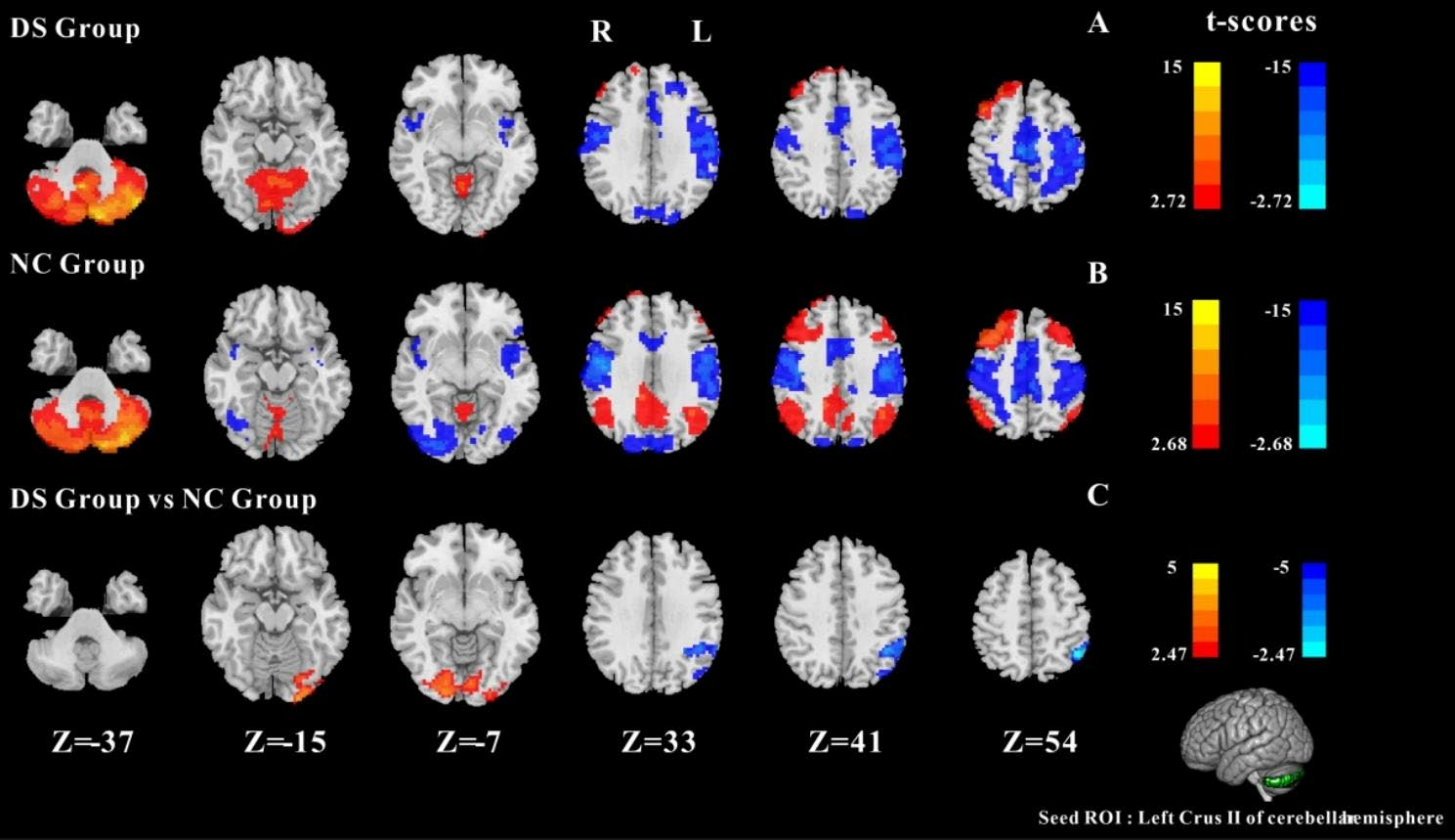
One-sample t-test results of FC based on left Crus II and whole cerebral voxels in the DS group (**A**) and in the NC group (**B**). The two-sample t-test results of FC based on left Crus II and whole cerebral voxels in the two groups (**C**); Note: the statistical map threshold was set at z > 2.32, with voxel correction at p < 0.01 and cluster correction at p < 0.05, GRF corrected for z-statistics. In one-sample t-test result, warm colors represent positive FC and cool colors represent negtive FC; In two-sample t-test result, warm colors show increased FC and cool colors show decreased FC. The color bar represents t-values. R: right, L: left

The results of two groups of one-sample t-test are shown in Fig. 2A and B. For the DS and NC, Crus I has significant FC with cerebellum, and significant negative FC with somatomotor Network. In addition, in the NC group, there was a significant FC between Crus II and the default mode network (bilateral PCUN, bilateral ANG, posterior cingulate gyrus), and there was a significant negative FC between Crus II and the visual area. The two-sample t-test results of the two groups are shown in Fig. 2C. The DS group showed significantly decreased FC between Crus II and left IPG and left ANG. Additionally, decreased negative FC between Crus II and the visual areas (left MOG, L-IOG., bilateral LING). (GRF corrected, cluster *p*-value < 0.05,voxel *p*-value<0.01).

#### Results of FC between Lobule IX of vermis and whole cerebral voxels

**Fig. 3:**
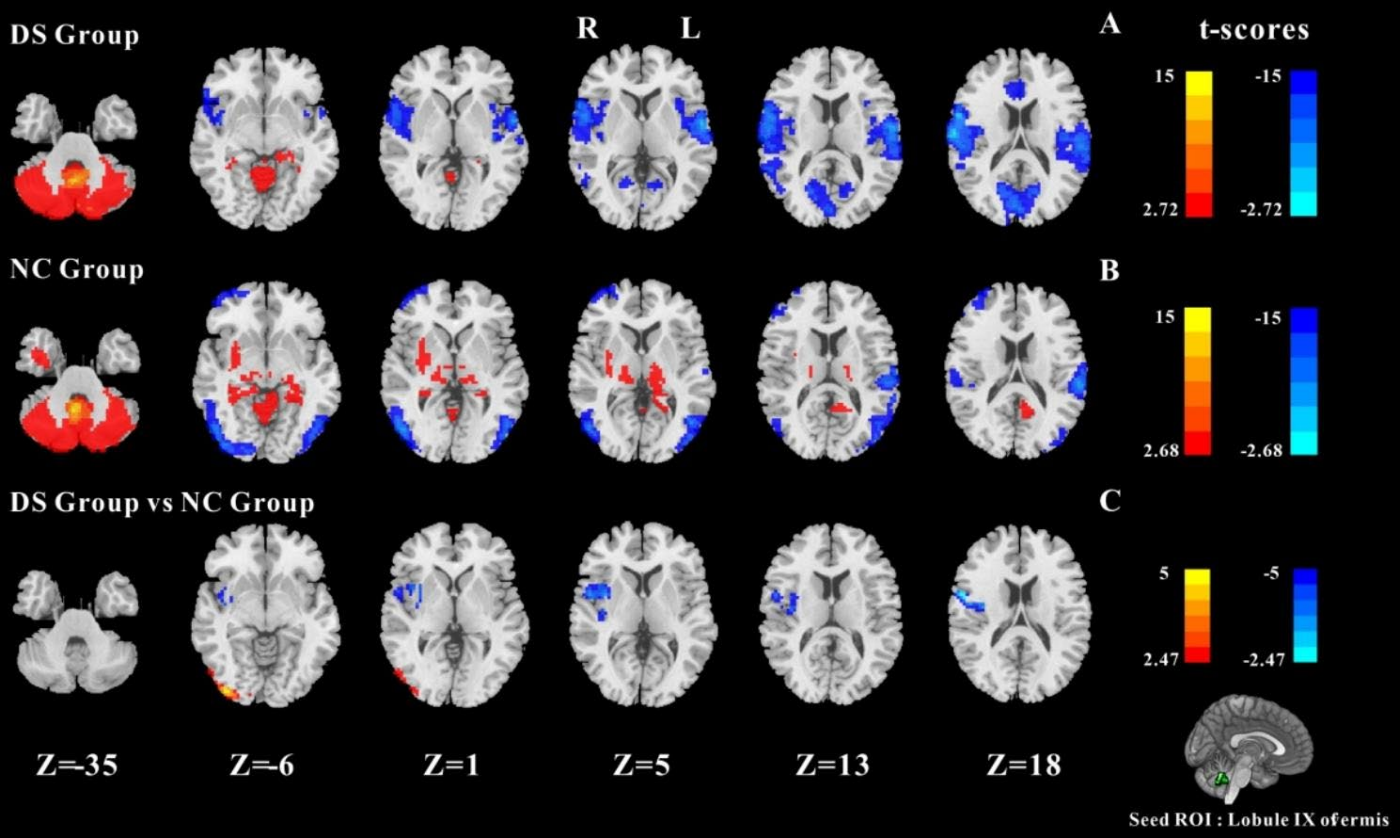
One-sample t-test results of FC based on left Lobule IX and whole cerebral voxels in the DS group (**A**) and in the NC group (**B**). The two-sample t-test results of FC based on left Lobule IX and whole cerebral voxels in the two groups (**C**); Note: the statistical map threshold was set at z > 2.32, with voxel correction at p < 0.01 and cluster correction at p < 0.05, GRF corrected for z-statistics. In one-sample t-test result, warm colors represent positive FC and cool colors represent negtive FC; In two-sample t-test result warm colors show increased FC and cool colors show decreased FC. The color bar represents t-values. R: right, L: left

The results of two groups of one-sample t-test t are shown in Fig. 3A and B. In the NC group, the Lobule IX had significant FC with the deep subcortical nuclei (including bilateral thalamus (THA), bilateral putamen, etc.) and negative FC with the bilateral occipital gyrus. The two-sample t-test results of the two groups are shown in Fig. 3C. Compared with the NC group, the DS group displayed decreased FC between Lobule IX and right PreCG, right INS, and right ROL. The negative FC between Lobule IX and visual areas (right OG, right LING) was decreased (GRF corrected, cluster p value < 0.05,voxel *p-*value<0.01).

#### Results of FC between Lobule X of vermis and whole cerebral voxels

**Fig. 4:**
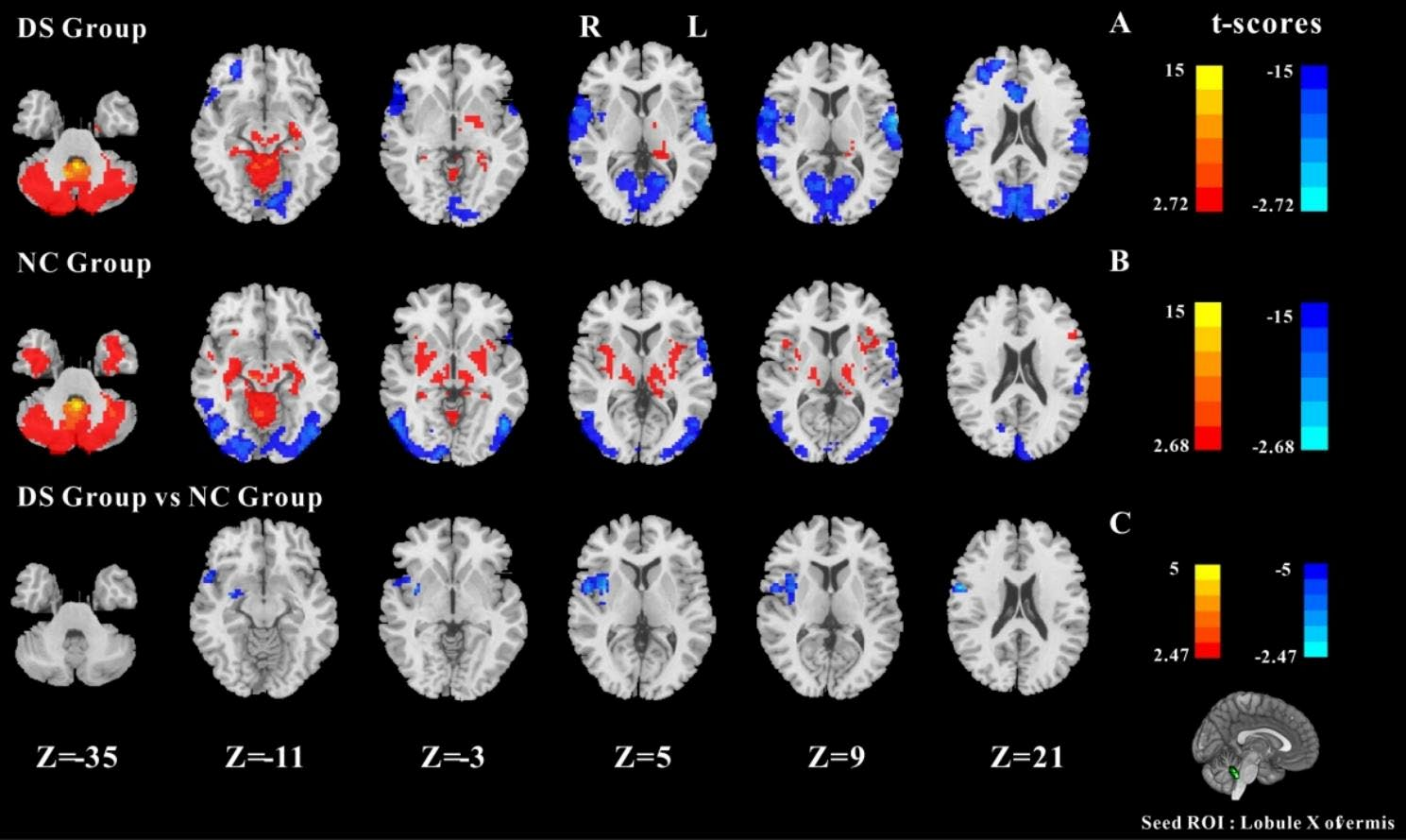
One-sample t-test results of FC based on left Lobule X and whole cerebral voxels in the DS group (**A**) and in the NC group (**B**). The two-sample t-test results of FC based on left Lobule X and whole cerebral voxels in the two groups (**C**); Note: the statistical map threshold was set at z > 2.32, with voxel correction at p < 0.01 and cluster correction at p < 0.05, GRF corrected for z-statistics. In one-sample t-test result, warm colors represent positive FC and cool colors represent negtive FC; In two-sample t-test result, warm colors show increased FC and cool colors show decreased FC. The color bar represents t-values. R: right, L: left

The results of two groups of one-sample t-test are shown in Fig. 4A and B. In the two groups, Lobule X had a significant negative FC with the somatomotor network and a strong FC with the cerebellar interior. In the NC group, there was a significant FC between the Lobule X and the deep subcortical nuclei (including bilateral THA, bilateral striatum, bilateral INS, and so on), and a significant negative FC between the Lobule X and the bilateral MOG. The DS group had negative FC between Lobule X and bilateral ROL and bilateral CUN. The two-sample t-test results of the two groups are shown in Fig. 4C. Compared to the NC group, the FC between the Lobule X and the right PreCG, right INS, and right ROL were decreased in the DS group (GRF corrected, cluster *p*-value = 0.05, voxel *p-*value<0.01).

### Correlation analysis

**Fig. 5:**
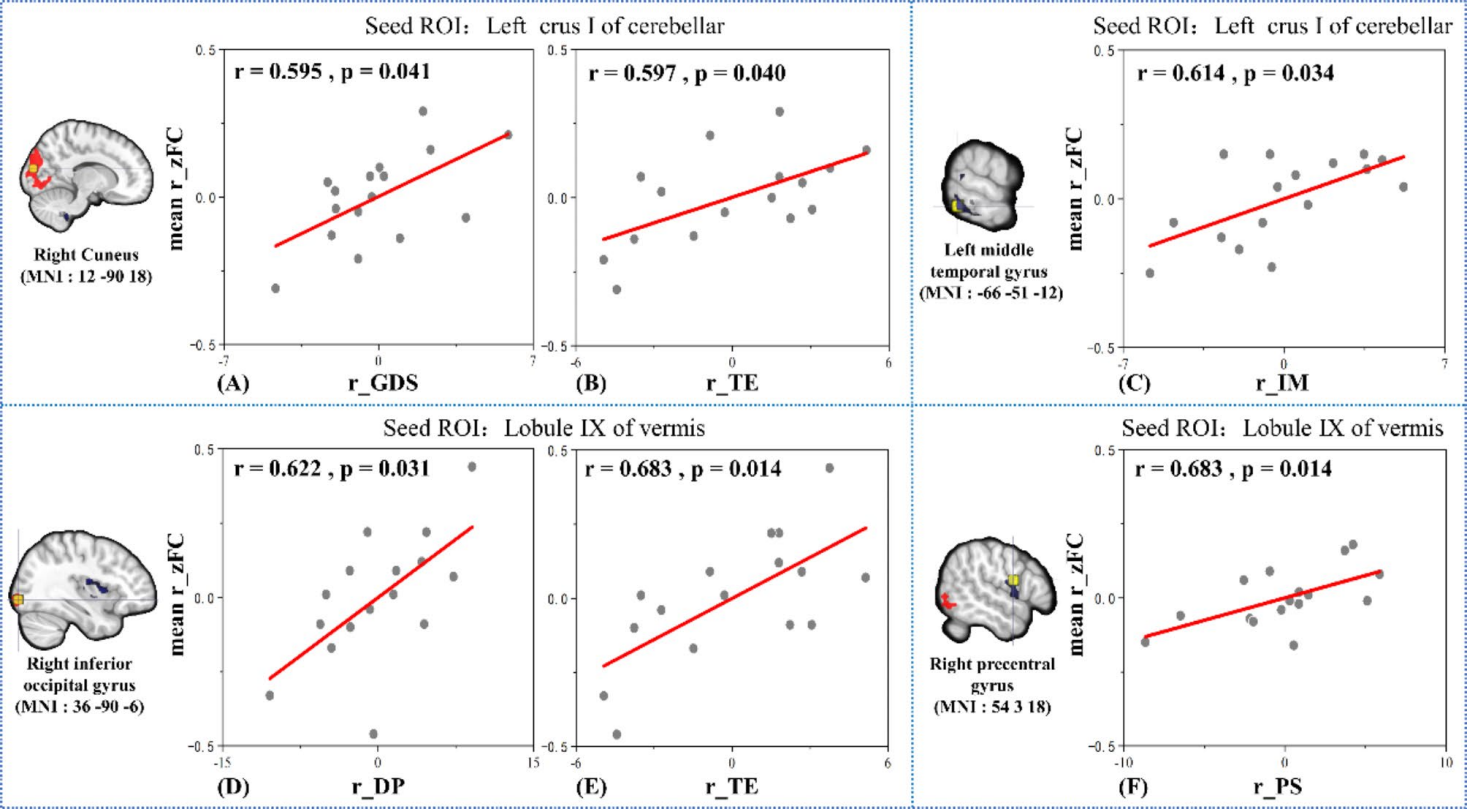
The results of correlation analysis in the DS group. Correlation analysis of FC between Crus I and right cuneus with GDS scores (**A**) and TE scores (**B**); Correlation analysis of FC between Crus I and left MTG with IM scores (**C**); Correlation analysis of FC between Crus Lobule IX and right IOG with DP scores (**D**) and TE scores (**E**); Correlation analysis of FC between Crus Lobule IX and right PreCG with PS scores (**F**). Note: The prefix ‘r’ represents the residual value after regressing for covatiates such as age, years of education, brain volume, and mean head movement. Abbreviations: PS: Problem-solving; DP: Death preparation; IM: Intimacy maintenance; TE: Teaching

The results of the correlation analysis in the DS group are shown in Fig. 5.GDS (r = 0.595,*p* = 0.041) and TE (r = 0.597,*p* = 0.040) were positively correlated with FC between left Crus I and right CUN (Fig. 5A and B). In addition, The negative FC between Crus I and the left MTG (MNI:-66 -51 -12) was positively correlated with IM (r = 0.614,*p* = 0.034)(Fig. 5C). The FC was positively correlated with DP (r = 0.622,p = 0.031) and TE (r = 0.683,p = 0.014) between Lobule IX and the right IOG (MNI:36–90 -6) (Fig. 5D and E). The negative FC between lobe IX and the right PreCG (MNI:54,318) had a positive correlation with PS (r = 0.603,p = 0.038) (Fig. 5F).

## Discussion

Several studies have demonstrated that the cerebellum is functionally connected to various brain structures through the cerebellar-thalamo-cortical circuit and plays an important role in higher functions such as cognition and emotion [43]. In this study, 26 sub-regions of the cerebellum were selected as ROI to calculate their FC with the whole cerebral region, then the difference in FC between the DS group and the NC group was compared, and finally, the correlation between FC of different cerebral regions and GDS score and RFS score was analysed.

### Analysis of cerebral regions with FC reduced the DS group compared to the NC group

The SFG and MFG are the main components of the dorsolateral prefrontal cortex (DLPFC), which is responsible for cognitive and executive functions and plays an important role in emotion regulation. In this study, the FC was reduced between left Crus I and left SFG and MFG in the DS group, the results were similar to those of previous studies [27, 44]. A study has found that patients with depression show low connectivity of the DLPFC when performing control tasks, suggesting that the cognitive executive capacity of the DLPFC is reduced and unable to inhibit negative emotional processing [45–47]. Thus, it is possible that the reduced FC in this study is associated with impaired emotional regulation and cognition in older patients with depression.

The SMG is part of the ventral parietal attention network and is involved in the attentional process of emotion management [48]. Our study found that the FC between the left Crus I and the left SMG was reduced in the DS group, which may be related to the decline in attentional function in older people with DS. A previous study found that the IPG lacked the activation of sad faces in patients with depression, which may be related to the decline of cognitive execution [49]. Compared with the NS group, the present study found that the FC between the left IPG and the left Crus I, II were reduced in the DS group. In addition, FC between Crus II and the left ANG was reduced in the DS group, a study found that FC of the ANG was reduced, which may be related to cognitive execution failure [50]. Based on the above research, we hypothesized that older women with DS may have cognitive processing impairments with negative emotions, which may be related to the onset of DS.

The occipital lobe is the core region for the processing of visual information, in particular, the processing of emotional faces [51]. The correct processing of facial information plays an important role in the maintenance of normal social functioning. Many previous studies have shown that occipital lobe activity or FC is significantly abnormal in patients with depression [52, 53]. In this study, negative FC was reduced between Crus I, II, Lobule IX and several cerebral regions of the occipital lobe. It has been proposed that positive FC in the rs-fMRI may indicate functional synergism, while negative FC may indicate functional antagonism [54]. In the present study, the negative FC between cerebellum and occipital lobe decreased, which may indicate the decreased antagonism between the cerebellum and the occipital gyrus. Therefore, we hypothesized that some cerebellar regions may be involved in the compensatory mechanism of visual cognitive processing in older women with DS. In addition, The FC between Crus I and the right CUN was positively correlated with the GDS score in the DS group. This may indicate that the CUN is an important cerebral region of the cerebellum involved in emotion regulation mechanisms.

The temporal lobe is involved in the processing of auditory information, facial expression recognition and other functions, and has an important influence on higher neural activities such as memory and emotion. A previous study reported abnormal neuronal activity in the temporal cortex of older patients with depression [55]. We find that compared to the NC group, the FC between Crus I and left MTG, left ITG was reduced in the DS group, similar to previous research results [28]. Based on the above research, we hypothesised that negative emotions might be related to depressive symptoms occurring.

The INS evaluates internal and external stimuli and plays an important role in emotion perception. A previous study showed that FC in the right INS correlated with the severity of DS in depressed patients [56]. The results of our study found that the FC between the right INS and Lobule IX was reduced in the DS group, which may be related to the decrease in positive emotional perception in older women with DS.

In addition, we also found that the FC was reduced, we also found a reduction in the FC between the right ROLwith Lobule IX and Lobule X, and the FC between Lobule IX and right PreCG was reduced, however, there are still few studies on the mechanism of the ROL and PreCG in depression, which need to be further explored.

### Analysis of cerebral regions with FC increased in the DS group compared to the NC group

This study only found that internal FC was enhanced in the Crus I regions of the DS group.

*It has been previously established that the cerebellum is involved in human higher-level cognitive and emotional processing* [57, 58], *with Crus I being one of the key brain regions implicated in cognitive-emotional integration* [59]. *A study on the dissociation of cognitive and motor functions in the cerebellum showed that during increased cognitive load, Crus I engages in activation processes with the prefrontal cortex, a pivotal node for controlling advanced cognitive functions* [60]. *Naismith et al. in their task-based fMRI research demonstrated that the cerebellum is among the primary brain regions activated in patients with depressive disorders* [61]. *Therefore, taking into consideration the aforementioned research findings, we hypothesize that the observed increase in FC in this study might be associated with excessive processing of negative emotions.*

### Correlation between abnormal FC and reminiscence functions scores in the DS group

Reminiscence functions refer to the role of individuals in the process of recalling past life experiences [62]. A previous study showed that positive reminiscences can help reduce negative emotions such as depression and anxiety [35]. However, negative reminiscences are more likely to be associated with depression in older people [63]. This study found that BD, IM and BR scores were higher in the DS group than in the NC group, and the difference in BR scores were statistically significant. BD, IM and BR all belong to the negative memory functions, the common feature is the immersion in repeated meditation on the past and the difficulty in accepting the unresolved events of the past [33]. Consistent with previous research, the results of this study suggest that the DS group is more likely to have negative reminiscences.

*Previous research had already confirmed a significant impact of autobiographical memory on late-life depression* [64, 65]. *An fMRI study demonstrated that as the severity of late-life depressive symptoms increases, the preference for positive memories decreases, and this memory bias affects the depressive symptoms in the elderly* [66]. *Correlation analyses in this study indicated that the FC strength between the left MTG, right CUN, right IOG right PreCG, and the cerebellum is positively correlated with scores on IM, PD, PS, and TE within reminiscence functions. This involves multiple memory and emotion-related brain areas, suggesting that reminiscence function was related to the mechanism of cognitive emotion regulation in the cerebellum. However, current research mainly focuses on the impact of autobiographical memory on late-life depression, and there is relatively limited investigation into the neural mechanisms of reminiscence functions in depressive symptoms. Further exploration is still needed*.

*This study still has limitations. first, the sample size of this study is small, which may affect the results of the study, so the sample size should be expanded in subsequent studies. Second, in this study, only GDS scores were used to distinguish DS group and NC group in older women, which should be combined with the evaluation of professional psychologists in future studies.*

## Conclusion

In conclusion, our study found abnormal functional connectivity between the cerebellum and several cerebral regions in the older women with DS group, involving the frontoparietal network, visual network, right INS, right ROL, right PreCG, and so on. At present, there are few studies on the involvement of cerebellum in DS in older women, this study provides a reference for future research on the neural mechanism of DS in older women.

### Electronic supplementary material

Below is the link to the electronic supplementary material.

Supplementary Material 1: Cerebral FC differences (DS vs. NC) using cerebellar regions as Seed ROIs.

## Data Availability

The datasets generated and/or analyzed during the current study are not publicly available due to privacy and ethical restrictions but are available from the corresponding author on reasonable request.

## Abbreviations

DS: Depressive symptoms
rs-fMRI: Resting-state functional magnetic resonance imaging
FC: Functional connectivity
NC: Normal controls
ROI: Regions of interest
GDS: Geriatric Depression Scale
RFS: Reminiscence Functions Scale
INS: Insula
ROL: Rolandic operculum
PreCG: Precentral gyrus
CUN: Cuneus
MTG: Maiddle temporal gyrus
PS: Problem-solving
ID: Identity
DP: Death preparation
BR: Bitterness revival
IM: Intimacy maintenance
TE: Teaching
BR: Boredom reduction
BOLD: Blood oxygen level-dependent
EPI: Echo Planar Imaging
FOV: Field of view
MNI: Montreal Neurological Institute
GRF: Gaussian random field
IPL: Inferior parietal gyrus, excluding supramarginal and angular gyri
SFG: Superior frontal gyrus
LING: Lingual gyrus
IOG: Inferior occipital gyrus
PCUN: Precuneus
ANG: Angular gyrus
IPG: Inferior parietal gyrus
SMG: Supramarginal gyrus
MFG: Middle frontal gyrus
ITG: Inferior temporal gyrus
MOG: Middle occipital gyrus
THA: Thalamus
DLPFC: Dorsolateral prefrontal cortex
