# Supplementary material for: Resting-state functional connectivity of the cerebellum-cerebrum in older women with depressive symptoms
Source: BMC Psychiatry. 2023 Oct 10;23:732. doi: 10.1186/s12888-023-05232-7 (PMC10566116; doi:10.1186/s12888-023-05232-7)
Supplement: Supplementary file 1 — Supplementary Material 1: Cerebral FC differences (DS vs. NC) using cerebellar regions as Seed ROIs. [file 12888_2023_5232_MOESM1_ESM.docx]

Table1 Cerebral FC differences (DS vs. NC) using cerebellar regions as Seed ROIs

| Anatomical description | AAL3 label | Peak t-value of cluster | MNI coordinates of peak t-value | | | Cluster size（voxels） |
| --- | --- | --- | --- | --- | --- | --- |
|  |  |  | X | Y | Z |  |
| **ROI：Left Crus I of cerebellar ( AAL3 Label: 95 )** | | | | | | |
| Right Cuneus (CUN) | 50 | 4.16 | 12 | -90 | 18 | 1126 |
| Left Inferior parietal gyrus, excluding supramarginal and angular gyri (IPG) | 65 | -5.31 | -60 | -42 | 45 | 479 |
| Left Superior frontal gyrus,  dorsolateral (SFG) | 3 | -3.88 | -15 | 36 | 33 | 353 |
| Left Middle temporal gyrus (MTG) | 89 | -5.03 | -66 | -51 | -12 | 247 |
| Right Lobule IX of cerebellar  hemisphere (CER9) | 110 | -4.48 | 6 | -45 | -45 | 200 |
| **ROI：Right Crus I of cerebellar (AAL3 Label: 96 )** | | | | | | |
| Right Supplementary motor area (SMA) | 16 | -4.36 | 6 | -21 | 51 | 139 |
| **ROI：Left Crus Ⅱ of cerebellar (AAL3 Label: 97 )** | | | | | | |
| Left Lingual gyrus (LING) | 51 | 4.17 | -21 | -99 | -15 | 604 |
| Left Inferior parietal gyrus,  excluding supramarginal and  angular gyri (IPG) | 65 | -4.65 | -45 | -54 | 54 | 399 |
| **ROI：Right Crus Ⅱ of cerebellar (AAL3 Label: 98 )** | | | | | | |
| Right Middle temporal gyrus (MTG) | 90 | 4.78 | 54 | -75 | 0 | 340 |
| Left Lobule VIIB of cerebellar  hemisphere (CER7b) | 105 | -4.57 | -39 | -63 | -48 | 262 |
| **ROI：Left Lobule III of cerebellar (AAL3 Label: 99 )** | | | | | | |
| - | - | - | - | - | - | - |
| **ROI：Right Lobule III of cerebellar (AAL3 Label: 100 )** | | | | | | |
| Right Middle temporal gyrus (MTG) | 90 | 5.73 | 57 | -69 | -3 | 264 |
| **ROI：Left Lobule IV, V of cerebellar (AAL3 Label: 101 )** | | | | | | |
| Left Lingual gyrus (LING) | 51 | 7.86 | -33 | -93 | -15 | 410 |
| **ROI：Right Lobule IV, V of cerebellar (AAL3 Label: 102 )** | | | | | | |
| - | - | - | - | - | - | - |
| **ROI：Left Lobule VI of cerebellar (AAL3 Label: 103 )** | | | | | | |
| Left Lingual gyrus (LING) | 51 | 4.48 | -27 | -90 | -18 | 188 |
| Left Inferior frontal gyrus,  opercular part (IFGoperc) | 7 | -3.94 | -36 | 3 | 21 | 165 |
| Left Inferior parietal gyrus,  excluding supramarginal and  angular gyri (IPG) | 65 | -3.28 | -42 | -51 | 48 | 146 |
| **ROI：Right Lobule VI of cerebellar (AAL3 Label: 104 )** | | | | | | |
| - | - | - | - | - | - | - |
| **ROI：Left Lobule VIIB of cerebellar (AAL3 Label: 105 )** | | | | | | |
| Left Middle occipital gyrus (MOG) | 55 | 5.58 | -42 | -81 | 0 | 498 |
| **ROI：Right Lobule VIIB of cerebellar (AAL3 Label: 106 )** | | | | | | |
| Right Calcarine fissure and  surrounding cortex (CAL) | 48 | 4.95 | 21 | -99 | 0 | 441 |
| Left Superior temporal gyrus (STG) | 85 | 6.09 | -48 | -42 | 12 | 170 |
| **ROI：Left Lobule VIII of cerebellar (AAL3 Label: 107 )** | | | | | | |
| Left Middle occipital gyrus (MOG) | 55 | 6.25 | -39 | -84 | -3 | 545 |
| **ROI：Right Lobule VIII of cerebellar (AAL3 Label: 108 )** | | | | | | |
| Left Olfactory_L (OLF) | 17 | 5.07 | -6 | 18 | -15 | 166 |
| **ROI：Left Lobule IX of cerebellar (AAL3 Label: 109 )** | | | | | | |
| Right Inferior occipital gyrus (IOG) | 58 | 4.17 | 36 | -90 | -6 | 175 |
| **ROI：Right Lobule IX of cerebellar (AAL3 Label: 110 )** | | | | | | |
| Left Fusiform gyrus (FFG) | 59 | -3.90 | -30 | -54 | -18 | 441 |
| Right Lingual gyrus (LING) | 52 | 4.12 | 27 | -99 | -18 | 276 |
| **ROI：Left Lobule X of cerebellar (AAL3 Label: 111 )** | | | | | | |
| Left Cuneus (CUN) | 49 | -4.62 | -12 | -75 | 18 | 247 |
| **ROI：Right Lobule X of cerebellar (AAL3 Label: 112 )** | | | | | | |
| - | - | - | - | - | - | - |
| **ROI：Lobule I, II of vermis (AAL3 Label: 113 )** | | | | | | |
| - | - | - | - | - | - | - |
| **ROI：Lobule III of vermis (AAL3 Label: 114 )** | | | | | | |
| Left Middle temporal gyrus (MTG) | 89 | -3.78 | -60 | -18 | 0 | 136 |
| **ROI：Lobule IV, V of vermis (AAL3 Label: 115 )** | | | | | | |
| Left Superior occipital gyrus (SOG) | 53 | 4.32 | -6 | -84 | 45 | 451 |
| Left Lingual_L (LING) | 51 | 5.94 | -30 | -90 | -15 | 260 |
| Left SupraMarginal gyrus (SMG) | 67 | -4.54 | -48 | -42 | 24 | 166 |
| Left Inferior temporal gyrus (ITG) | 93 | -3.75 | -51 | -24 | -18 | 165 |
| **ROI：Lobule VI of vermis (AAL3 Label: 116 )** | | | | | | |
| Left Lingual gyrus (LING) | 51 | 4.42 | -24 | -90 | -15 | 227 |
| **ROI：Lobule VII of vermis (AAL3 Label: 117 )** | | | | | | |
| Left Lingual gyrus (LING) | 51 | 4.57 | -27 | -93 | -15 | 174 |
| **ROI：Lobule VIII of vermis (AAL3 Label: 118 )** | | | | | | |
| Left Lingual gyrus (LING) | 51 | 5.89 | -27 | -93 | -15 | 212 |
| Right Lingual gyrus (LING) | 52 | 4.49 | 18 | -96 | -9 | 207 |
| **ROI：Lobule IX of vermis (AAL3 Label: 119 )** | | | | | | |
| Right Precentral gyrus (PreCG) | 2 | -5.06 | 54 | 3 | 18 | 332 |
| Right Inferior occipital gyrus (IOG) | 58 | 4.47 | 36 | -90 | -6 | 216 |
| **ROI：Lobule X of vermis (AAL3 Label: 120 )** | | | | | | |
| Right Precentral gyrus (PreCG) | 2 | -5.20 | 57 | 3 | 21 | 349 |


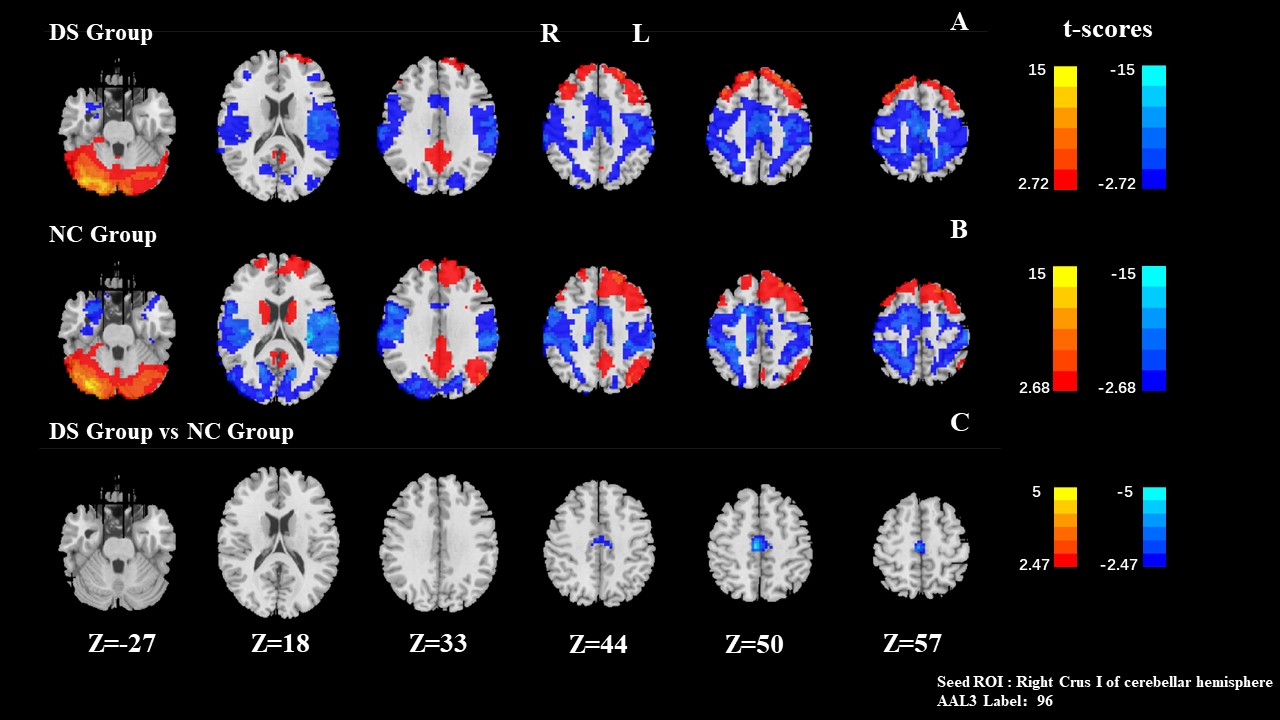


Figure 1. FC results of seed ROI: Right Crus I of cerebellar (AAL3 Label: 96 )


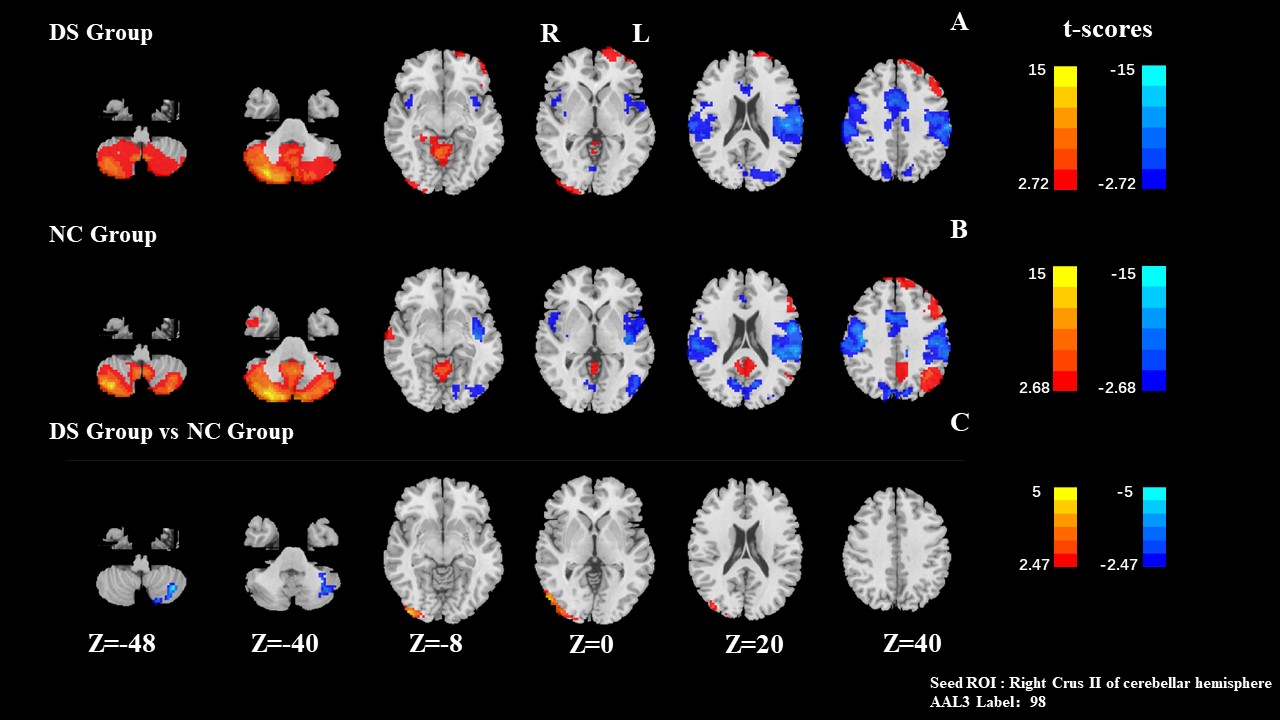


Figure 2. FC results of seed ROI: Right Crus Ⅱ of cerebellar (AAL3 Label: 98 )


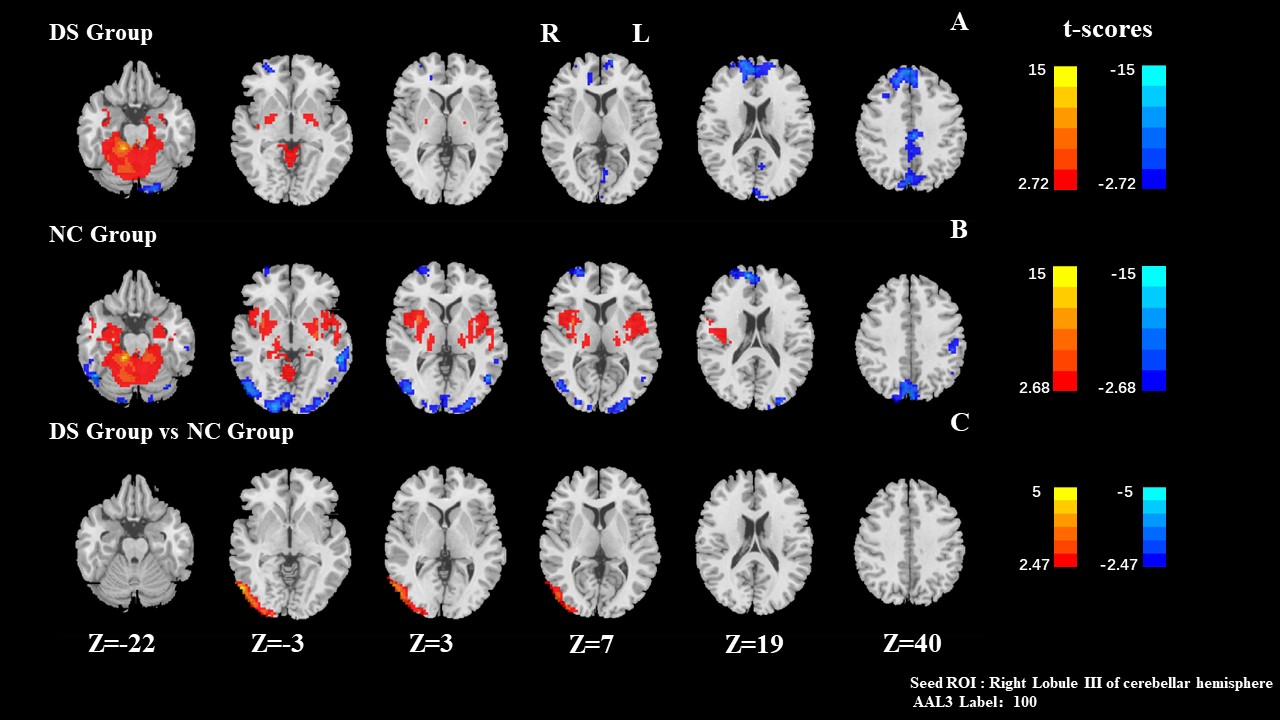


Figure 3. FC results of seed ROI: Right Lobule III of cerebellar (AAL3 Label: 100 )


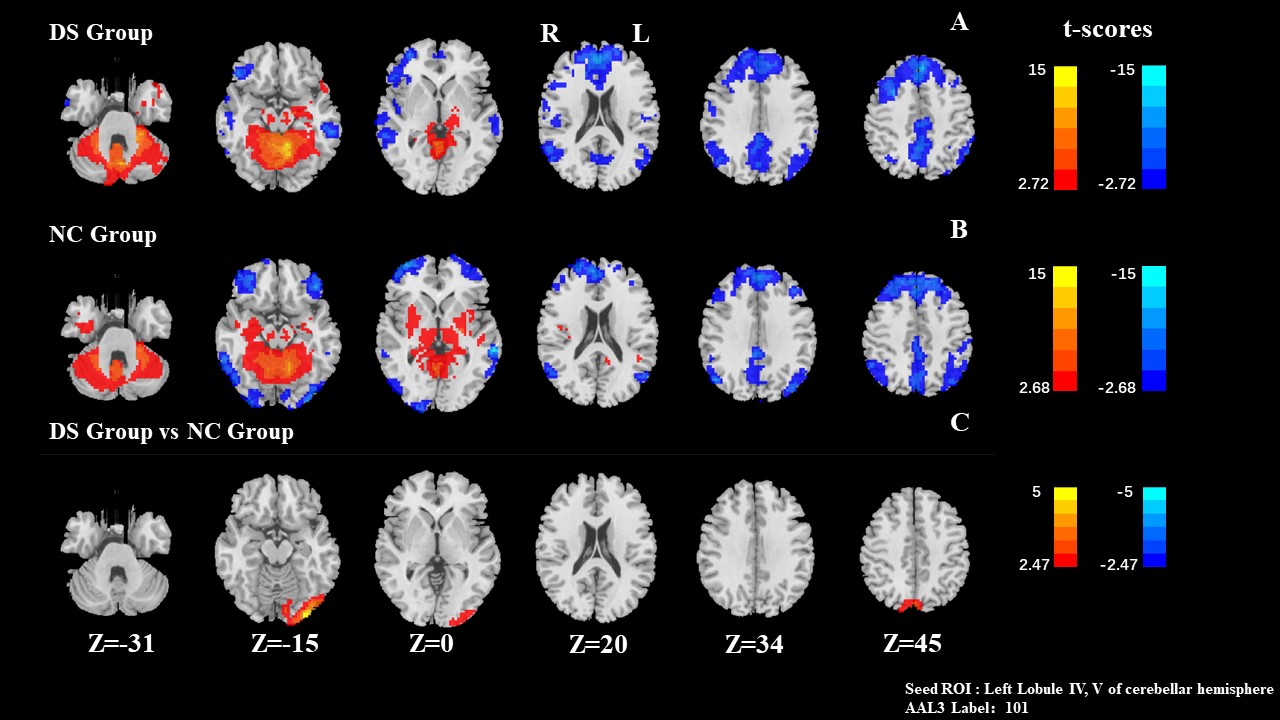


Figure 4. FC results of seed ROI: Left Lobule IV, V of cerebellar (AAL3 Label: 101 )


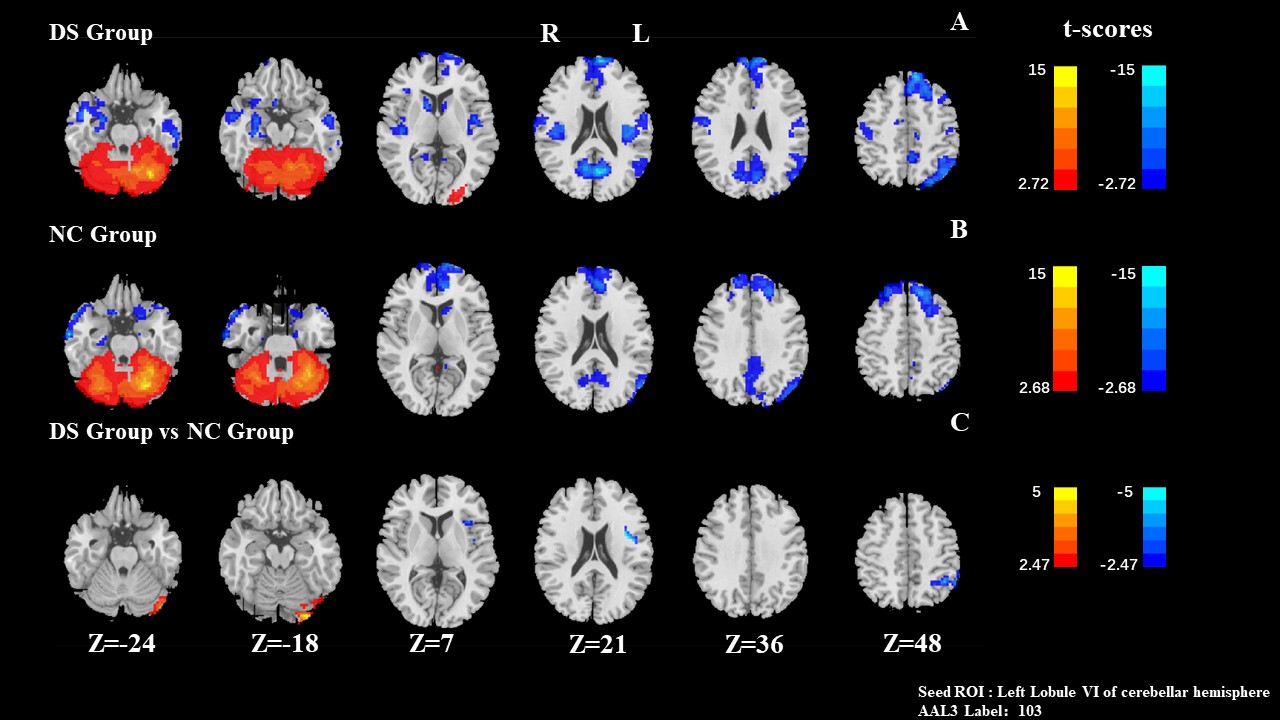


Figure 5. FC results of seed ROI: Left Lobule VI of cerebellar (AAL3 Label: 103 )


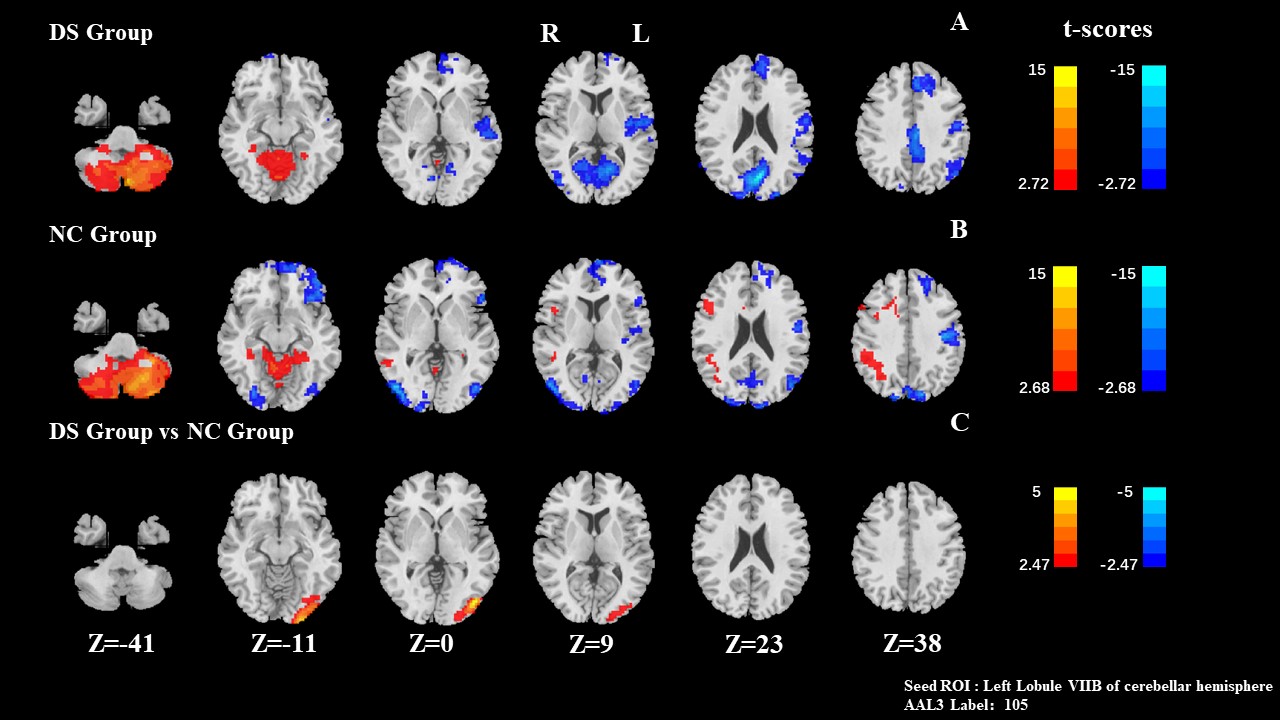


Figure 6. FC results of seed ROI: Left Lobule VIIB of cerebellar (AAL3 Label: 105 )


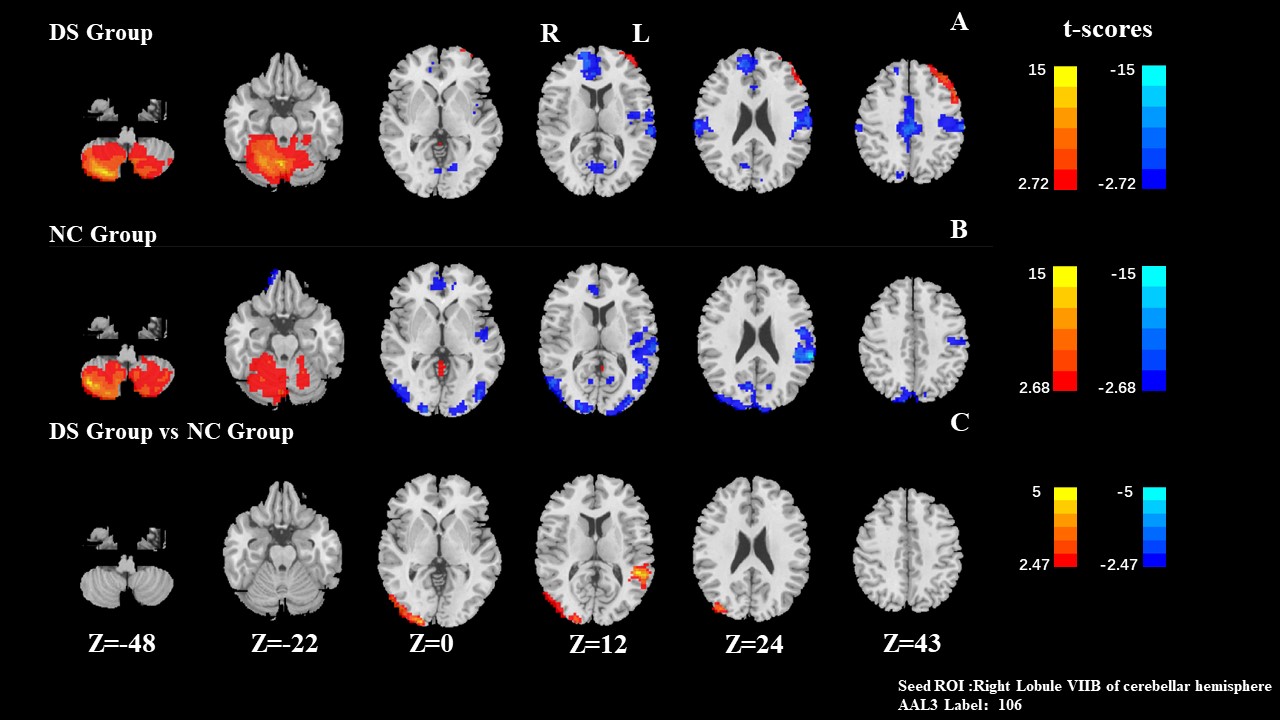


Figure 7. FC results of seed ROI: Right Lobule VIIB of cerebellar (AAL3 Label: 106 )


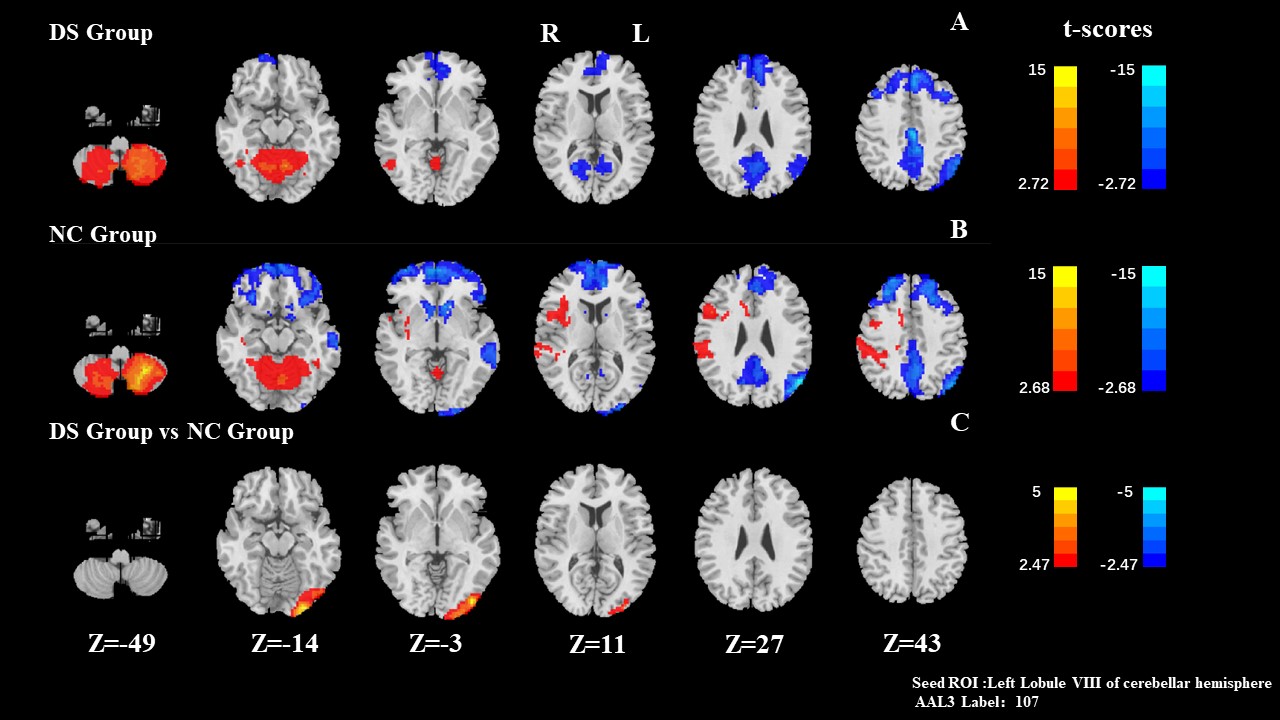


Figure 8. FC results of seed ROI: Left Lobule VIII of cerebellar (AAL3 Label: 107 )


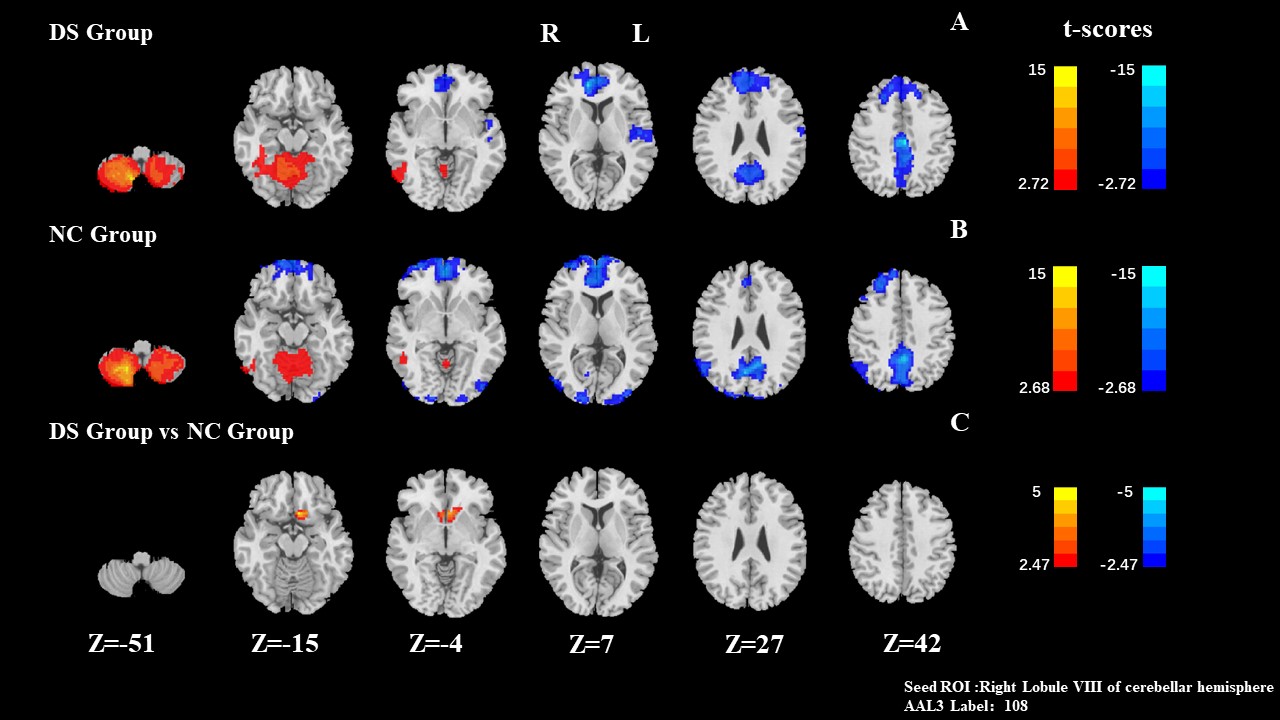


Figure 9. FC results of seed ROI: Right Lobule VIII of cerebellar (AAL3 Label: 108 )


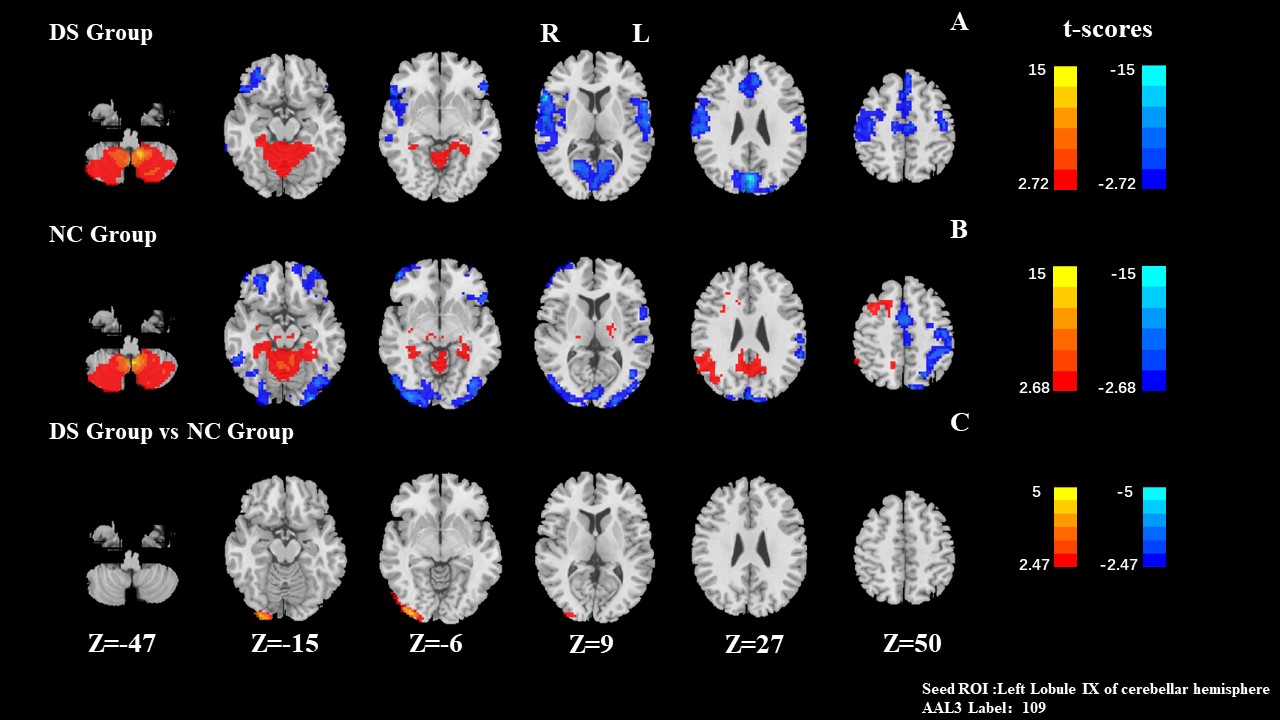


Figure 10. FC results of seed ROI: Left Lobule IX of cerebellar (AAL3 Label: 109 )


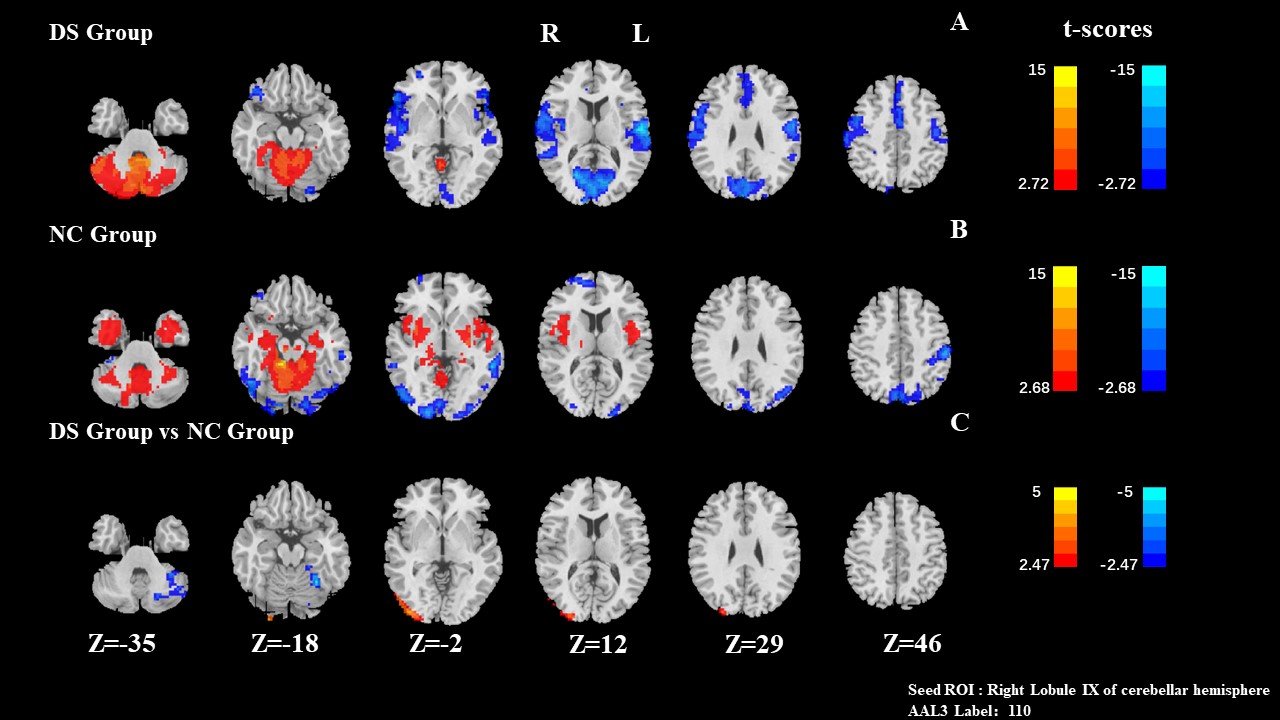


Figure 11. FC results of seed ROI: Right Lobule IX of cerebellar (AAL3 Label: 110 )


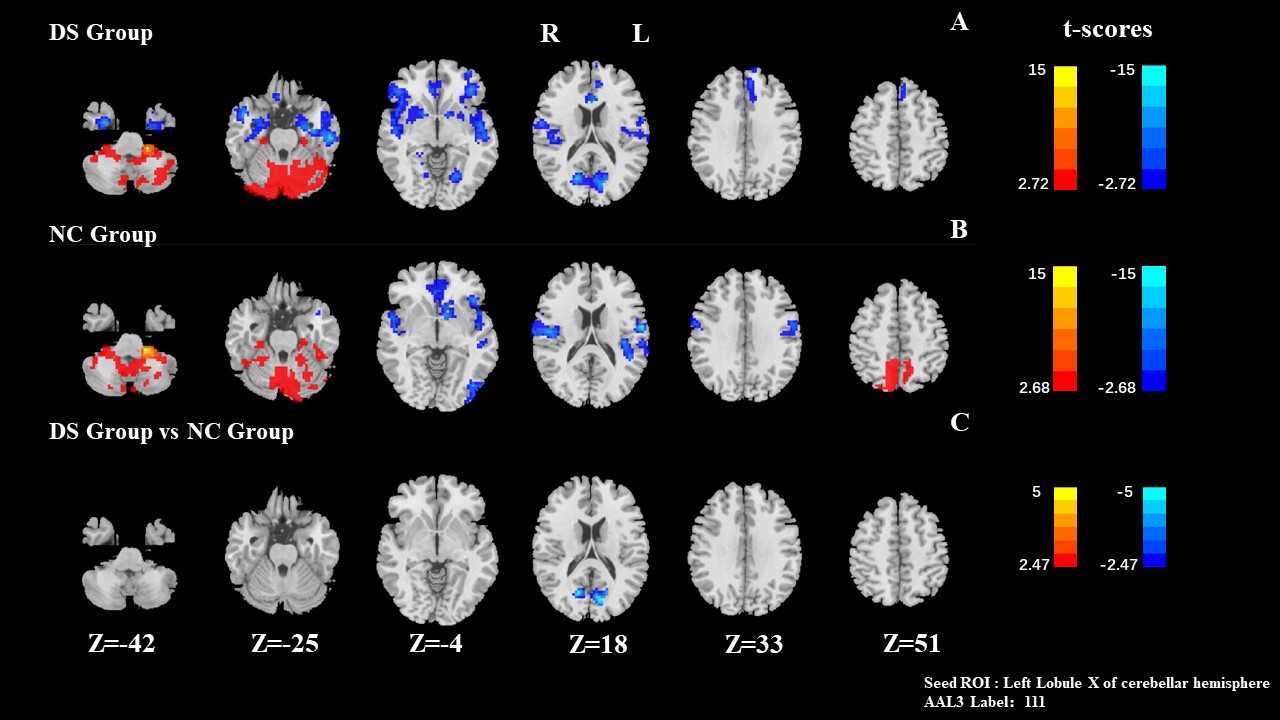


Figure 12. FC results of seed ROI: Left Lobule X of cerebellar (AAL3 Label: 111 )


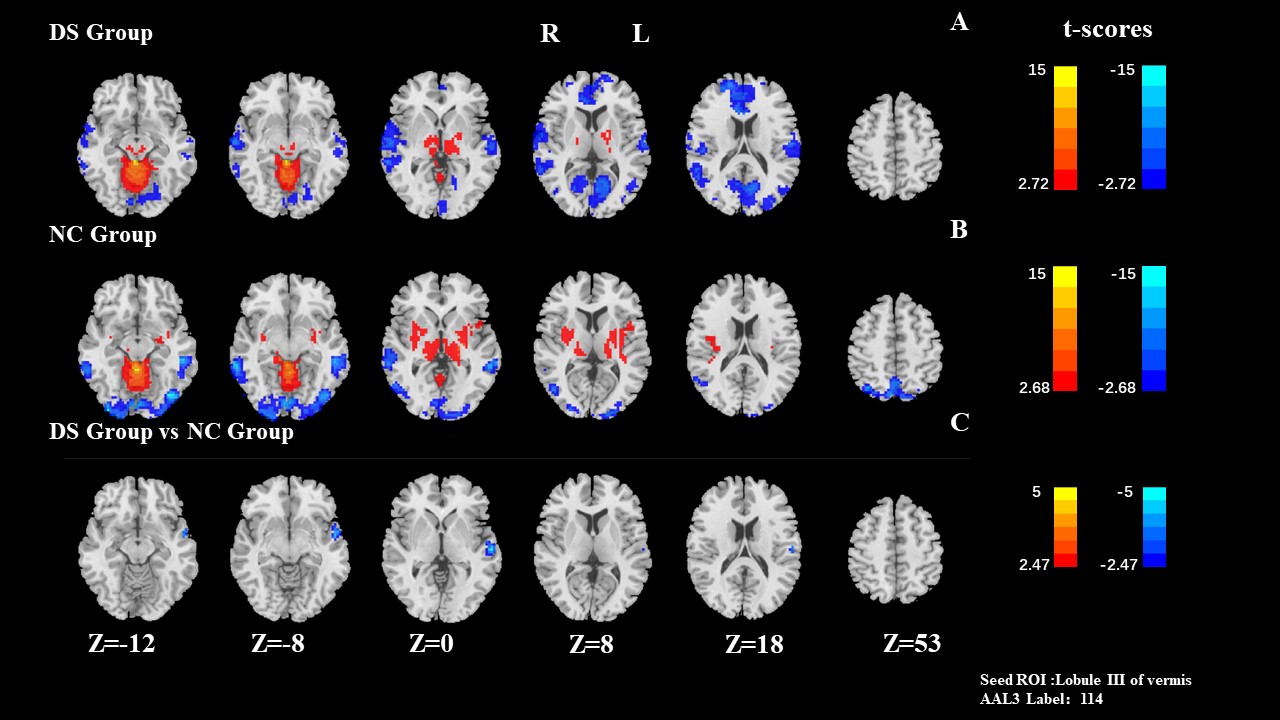


Figure 13. FC results of seed ROI: Lobule III of vermis (AAL3 Label: 114 )


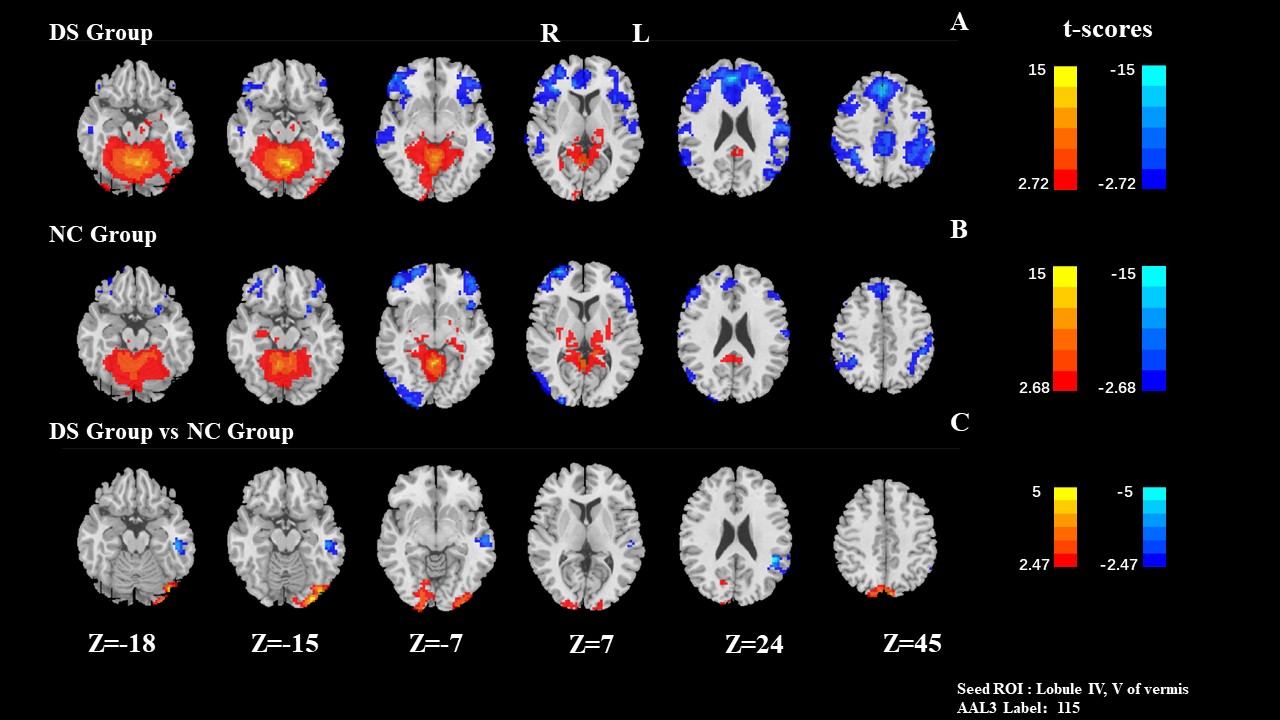


Figure 14. FC results of seed ROI: Lobule IV, V of vermis (AAL3 Label: 115 )


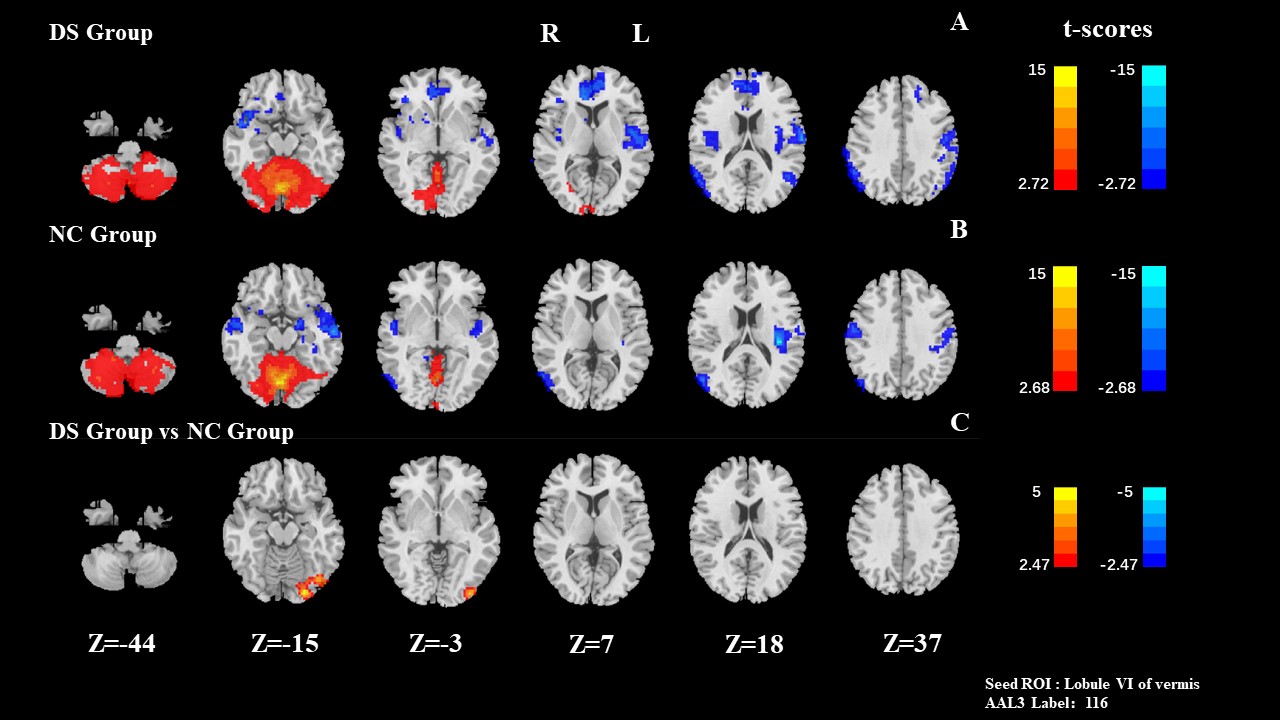


Figure 15. FC results of seed ROI: Lobule VI of vermis (AAL3 Label: 116 )


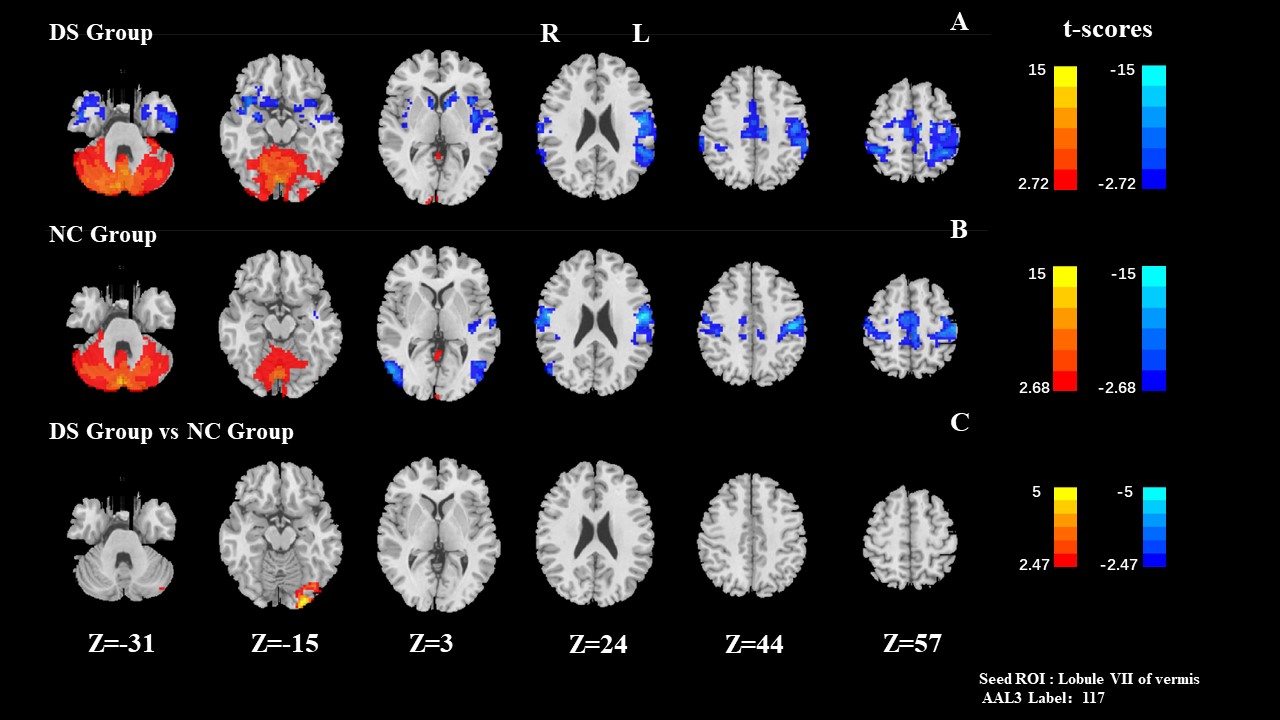


Figure 16. FC results of seed ROI: Lobule VII of vermis (AAL3 Label: 117 )


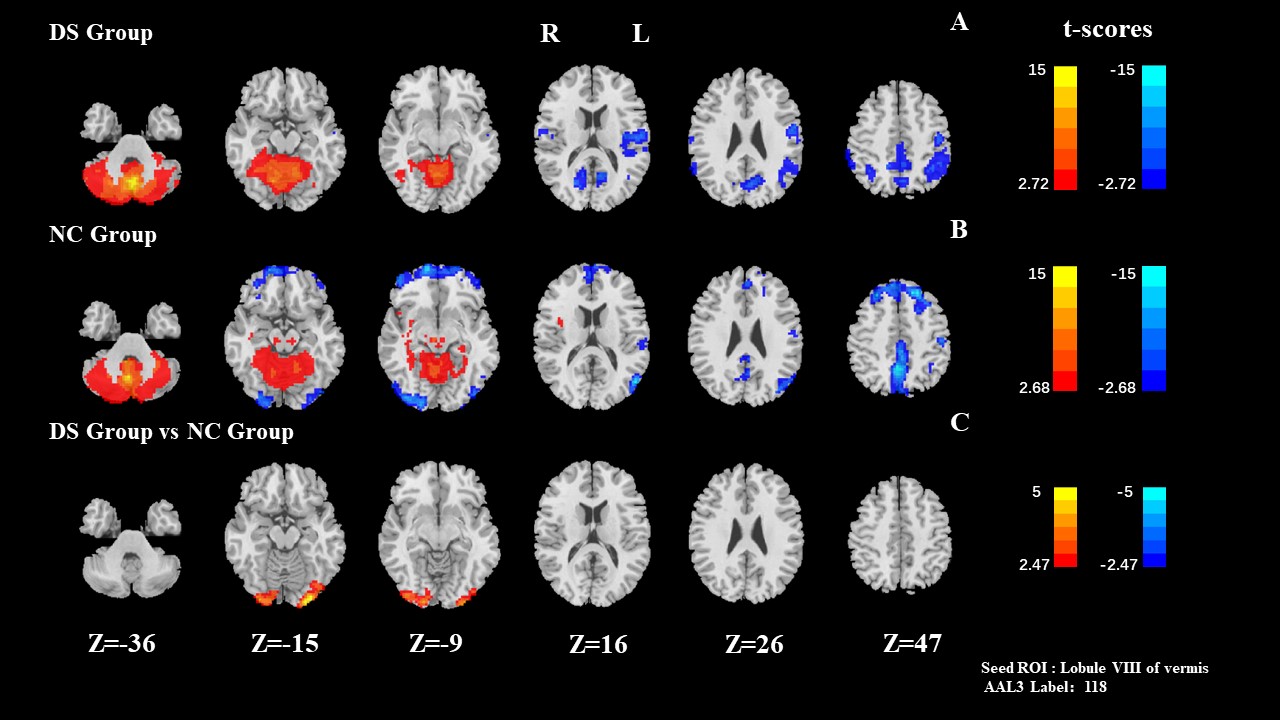


Figure 17. FC results of seed ROI: Lobule VIII of vermis (AAL3 Label: 118 )
